# Loss of functional peroxisomes leads to increased mitochondrial biogenesis and reduced autophagy that preserve mitochondrial function

**DOI:** 10.1007/s00018-023-04827-3

**Published:** 2023-06-20

**Authors:** Lijun Chi, Dorothy Lee, Sharon Leung, Guanlan Hu, Bijun Wen, Paul Delgado-Olguin, Miluska Vissa, Ren Li, John H. Brumell, Peter K. Kim, Robert H. J. Bandsma

**Affiliations:** 1 Translational Medicine Program, The Hospital for Sick Children, Peter Gilgan Centre for Research and Learning, 686 Bay Street, Toronto, ON M5G 0A4 Canada; 2 Department of Physiology, University of Toronto, Toronto, ON M5S 1A1 Canada; 3 Cell Biology Program, The Hospital for Sick Children, Peter Gilgan Centre for Research and Learning, 686 Bay Street, Toronto, ON M5G 0A4 Canada; 4 Department of Nutritional Sciences, University of Toronto, Toronto, ON M5S 1A1 Canada; 5 Department of Molecular Genetics, University of Toronto, Toronto, ON M5S 1A1 Canada; 6 Heart and Stroke Richard Lewar Centre of Excellence, Toronto, ON M5S 3H2 Canada; 7 Department of Biochemistry, University of Toronto, Toronto, ON M5S 1A1 Canada; 8 Centre for Global Child Health, The Hospital of Sick Children, Toronto, ON M5G 0A4 Canada; 9 Division of Gastroenterology, Hepatology and Nutrition, The Hospital for Sick Children, Toronto, ON M5G 0A4 Canada

**Keywords:** Mitophagy, Malnutrition, Fenofibrate, Nuclear hormone receptor, mTOR, Metabolism

## Abstract

Peroxisomes are essential for mitochondrial health, as the absence of peroxisomes leads to altered mitochondria. However, it is unclear whether the changes in mitochondria are a function of preserving cellular function or a response to cellular damage caused by the absence of peroxisomes. To address this, we developed conditional hepatocyte-specific *Pex16* deficient (*Pex16* KO) mice that develop peroxisome loss and subjected them to a low-protein diet to induce metabolic stress. Loss of PEX16 in hepatocytes led to increased biogenesis of small mitochondria and reduced autophagy flux but with preserved capacity for respiration and ATP capacity. Metabolic stress induced by low protein feeding led to mitochondrial dysfunction in *Pex16* KO mice and impaired biogenesis. Activation of PPARα partially corrected these mitochondrial disturbances, despite the absence of peroxisomes. The findings of this study demonstrate that the absence of peroxisomes in hepatocytes results in a concerted effort to preserve mitochondrial function, including increased mitochondrial biogenesis, altered morphology, and modified autophagy activity. Our study underscores the relationship between peroxisomes and mitochondria in regulating the hepatic metabolic responses to nutritional stressors.

**Supplementary Information:**

The online version contains supplementary material available at 10.1007/s00018-023-04827-3.

## Introduction

Hepatic peroxisomes are critical for the anabolic and catabolic processing of lipids, including the oxidation of very long-chain fatty acids and branched fatty acids, and the synthesis of plasmalogens, bile acids and sterol precursors. These lipid biochemical reactions are not entirely completed in peroxisomes as they require coordination with other organelles, including the ER and mitochondria [1, 2]. Peroxisomes are important for mitochondrial health, as genetic diseases such as Zellweger Spectrum Disorders, which result in the loss of functional peroxisomes, are associated with hepatic mitochondrial dysfunction [3, 4]. However, our understanding of the underlying mechanisms is limited.

Peroxisome loss in Zellweger Spectrum Disorders results from defects in one of 14 peroxins (PEX) responsible for peroxisome biogenesis and homeostasis [1, 5]. Some peroxins, such as PEX11, are essential for peroxisomal growth and division. Others, including PEX5, PEX7, PEX14, and PEX13 regulate peroxisomal matrix protein import, while PEX3, PEX16, and PEX19 are essential in initiating peroxisome biogenesis [5]. The loss of peroxins such as PEX5 results in ghost peroxisomes with membrane structures devoid of matrix proteins [6], whereas the loss of PEX16 completely abolishes peroxisomes [7]. Because peroxisomes interact with mitochondria [1, 2], the complete abolishment of peroxisomes may help us understand the functional interrelationship between these two organelles.

Mitochondria continuously undergo fission and fusion to maintain dynamic homeostasis. These processes are mediated primarily by Dynamin-related protein 1 (DRP1) and Mitofusin-2 (MFN2), respectively [8]. Impaired mitochondrial function can lead to reduced ATP production, excessive reactive oxygen species (ROS), altered metabolism, changes in calcium storage and apoptosis. The accumulation of damaged mitochondria increases cellular oxidative stress and the likelihood of cell death. Therefore impaired mitochondria are turned over by a selective autophagy called mitophagy [9–11].

Various peroxisome deficient mouse models have provided insight into hepatic mitochondrial homeostasis and function. PEX5 deficiency leads to the proliferation of mitochondria with altered morphology and function, and reduced respiration mainly due to a decrease in complex I [12, 13]. A similar phenotype was observed in PEX2 and PEX13 deficient mice [14, 15]. Similar mitochondria defects were also observed in metabolic stress conditions that cause peroxisome loss in the hepatocytes of non-PEX mutant animals. We recently reported that young mice and rats fed a very low protein diet had dramatically reduced hepatic peroxisomes due to the upregulation of peroxisome-specific autophagy, i.e., pexophagy. This reduction of peroxisomes was also associated with damaged and dysfunctional mitochondria [16, 17]. Interestingly, mitochondrial volume increases in most PEX deficient animal models and it has been suggested that this may be a consequence of increased mitochondrial biogenesis [11]. Alternatively, the accumulation of damaged and dysfunctional mitochondria may also result from defects in mitochondria quality control. Thus, it remains unclear whether these changes in mitochondria in hepatocytes without peroxisomes reflect an adaptive response to preserve cellular function or indicate cellular damage due to the loss of peroxisomes.

In this study, we aimed to uncover the role of peroxisomes in mitochondrial homeostasis. To understand the connection between peroxisome loss and mitochondrial function, we generated a liver-specific *Pex16* knockout mouse and analyzed mitochondrial morphology, dynamics, and function. These mice were studied under normal nutritional conditions (i.e., normal diet) and nutritional stress (i.e., low protein diet feeding). We found that the loss of PEX16 increased the formation of small mitochondria through the upregulation of genes controlling their fission and fusion. Surprisingly, autophagy flux was reduced in the peroxisome-deficient model, potentially due to a reduction in the degradation of damaged mitochondria. The nutritional stressor led to mitochondrial dysfunction associated with the repression of their biogenesis despite an increase in autophagy. Upregulating mitochondria biogenesis and β-oxidation through PPARα activation improved mitochondrial homeostasis and function independently of the presence of peroxisomes.

## Materials and methods

### Mouse generation

The Albumin-Cre *Pex16* flox/flox (Pex16f/f) mice were generated from Pex16tm1a(EUCOMM)Hmgu, which were obtained from the EUCOMM repository. The Pex16tm1a(EUCOMM)Hmgu were maintained on a C57BL/6J genetic background, and the *Pex16* flox/flox mice were generated by crossing with mice expressing Flp recombinase under an actin promoter. To generate the hepatocyte specific *Pex16* deficient mice (*Pex16* KO), *Albumin-Cre* males were crossed with *Pex16*^*f/f*^ females. Male *Pex16*^*f/*+^*;Alb-cre* mice were further crossed with female *Pex16*^*f/f*^ to generate homozygous *Pex16*^*f/f*^*;Alb-cre* (aka *Pex16* KO*)* and wild-type control mice. Only wild-type controls and homozygous *Pex16*^*f/f*^*;Alb-cre* males were used in the experiments. Genotypes were confirmed by PCR (Table S1). Procedures followed the Canadian Council for Animal Care guidelines and were approved by the Animal Care Committee at the Hospital for Sick Children.

### Low protein diet mouse model and fenofibrate treatment

*Pex16* KO and wild-type control mice were randomized at weaning (i.e., age 3 weeks) into two groups: (1) fed a normal 18% protein chow or (2) fed a low protein diet (LPD; 1% protein diet). The diets were isocaloric but differed in protein content (Fig. S2). Diets were continued for 14 days when animals were fasted for 5 h before being sacrificed. For the fenofibrate treatment experiments, *Pex16* KO and wild-type control mice were randomized at weaning into four groups (1) fed a normal 18% protein chow or (2) fed an 18% protein chow and receiving fenofibrate treatment, (3) fed LPD 1% protein diet and (4) LPD and receiving fenofibrate. Fenofibrate (Sigma, F6020) was dissolved in dimethyl sulfoxide (DMSO) (concentration of 100 μg/μL) and given by gavage at a dose of 100 µg/g body weight. Fenofibrate treatment started 7 days after initiation of dietary interventions and continued for 7 days until sacrifice. DMSO was used for the control vehicle group.

### Modulating autophagy flux

*Pex16* KO and wild-type control mice were fed their attributed diets for 2 weeks and chloroquine (CQ) (Abcam, Cambridge, UK) treatment was delivered by intraperitoneal (IP) injection with a dose of 50 mg/kg of body weight 8 h prior to sacrifice. Mice were fasted for 4 h before sacrifice, and livers were harvested for various assays.

### Histology and immunofluorescence

Livers were fixed, embedded in paraffin, sectioned (5 µm) and stained with H&E. Frozen sections (10 µm) were stained with Oil Red O or immunofluorescent antibodies. For this procedure, frozen sections were blocked with 3% bovine serum albumin (BSA) and 0.1% Tween-20 in phosphate-buffered saline (PBS) for 1 h at room temperature. Antibodies (Table S2) were diluted in 1% BSA and 0.1% Tween-20 in PBS. Slides were incubated in primary antibodies overnight at 4 °C, washed and then incubated with secondary antibodies (Table S2). Slides were stained with 4′,6-diamidino-2-phenylindole (DAPI) for 1 h at room temperature. Liver cryosections with 4-μm thickness were stained with 1:1000 fluorinated boron-dipyrromethene (BODIPY) to visualize fat droplets.

### Western blotting

In brief, livers were lysed through sonication of tissue with an extraction buffer and protease inhibitor cocktail (Sigma-Aldrich, St. Louis, MO, USA). Protein concentration was measured by Bicinchoninic Acid assay as per manufacturer’s instructions (Thermo Fisher Scientific, Waltham, MA, USA). Samples were then denatured by boiling in sodium dodecyl sulfate (SDS) and generally, 20 μg of protein was loaded onto SDS-PAGE gels. Proteins were transferred onto polyvinylidene fluoride membranes with Trans-Blot system (Thermo Fisher Scientific). Membranes were blocked with 3% BSA and 0.1% Tween-20 in tris-buffered saline (TBS) for one hour at room temperature. Antibodies (Table S2) were diluted in 1% BSA and 0.1% Tween-20 in TBS. Membranes were incubated in primary antibodies overnight at 4 °C, followed by secondary antibodies incubation for 1 h at room temperature. Membranes were visualized using Pierce enhanced chemiluminescence (Invitrogen, Carlsbad, CA, USA).

### qPCR

Total RNA was extracted from the liver using Direct-zol RNA Miniprep Kit (ZYMO Research Inc., USA). cDNA was synthesized from 500 ng of total RNA using the SuperScript VILO cDNA Synthesis Kit (Thermo Fisher Scientific, Rockford, IL, USA). For qPCR, 0.1 μl of cDNA was used with Advanced qPCR Mastermix with SuperG Dye (Wisent, Bioproducts, Saint-Jean-Baptiste, QC, Canada). mtDNA was measured using 500 ng of genomic DNA in each qPCR reaction. qPCR was performed using a CFX384 Touch Real-Time PCR Detection System (Bio-Rad Laboratories, Hercules, CA, USA) using the primers listed in Table S3.

### Hepatic triglyceride assay

Briefly, 50 mg of wet liver tissue was homogenized in cold PBS and tissue lipids were extracted with methanol-chloroform, dried and dissolved for triglyceride analysis. Liver and serum triglyceride concentrations were determined with a commercial kit (Randox Laboratories, Crumlin, UK). Photometric absorbances were read at 500 nm. Triglyceride values were normalized to protein concentration calculated from the same liver tissue by BCA assay as described above.

### Hepatic ATP assay

Liver tissue (~ 10 mg) was homogenized using a dounce homogenizer in perchloric acid (PCA). Excess PCA was precipitated by adding ice-cold 2 M KOH to neutralize the samples (pH 6.5–8). The deproteinized samples were then assayed. ATP concentrations were measured using a commercial ATP (Colorimetric/Fluorometric) assay kit (Abcam). Fluorescence was measured at Excitation/Emission = 537/578 nm with Skanlt software by Varioskan Lux Multimode Microplate Reader (Thermo Fisher Scientific). ATP concentration was established by comparing fluorescence readings from samples to standards provided within the kit.

### Transmission electron microscopy (TEM)

For transmission electron microscopy (TEM), fresh liver samples (~ 1 mm^2^) were fixed in 2% glutaraldehyde in 0.1 M sodium cacodylate buffer, rinsed in cacodylate buffer, post-fixed in 1% osmium tetroxide in cacodylate buffer. Samples were then dehydrated through a series of graded ethanol followed by incubation in propylene oxide, and then embedded in Quetol-Spurr resin. Sections (90 nm) were cut on a Leica Ultracut ultramicrotome (Leica Microsystem, Wetzlar, Germany), stained with uranyl acetate and lead citrate, and viewed in an FEI Tecnai 20 transmission electron microscope (Field Electron and Ion Company, Hillsboro, Oregon, USA). Mitochondrial morphology was measured using Image J software.

### Central carbon metabolism assay

To quantify metabolites of central carbon metabolism (CCM) in mouse liver, we used UPLC-MRM/MS, a Dionex 3400 UHPLC system (Dionex Sunnyvale, CA, USA) coupled to a 4000 QTRAP mass spectrometer (SCIEX, Framingham, MA, USA). Liver metabolites were extracted and quantified including those from tricarboxylic acid (TCA) cycle, glucose and selected sugar phosphates, and other phosphate-containing metabolites and nucleotides (Tables S4 and S5). Concentrations of the detected metabolites were calculated from internal linear-regression calibration curves.

### Isolation of liver mitochondria and mitochondrial respiration

Mitochondria were isolated from liver tissue by differential centrifugation as described previously [18]. The livers were dissected and minced in ice-cold mitochondrial isolation buffer composed of 100 mM Tris-MOPS, 10 mM EGTA/Tris and 90 mM sucrose, adjusted pH to 7.4. The suspension was transferred to a glass dounce homogenizer and homogenized. Following a series of centrifugations at different speeds at 4 °C, the pellet containing mitochondria was resuspended in an approximate volume in the mitochondrial isolation buffer. Total mitochondrial protein was determined using Pierce BCA Protein Assay kit (Thermo Fisher Scientific). Finally, the mitochondrial preparations (5 mg/well) were used for the coupling assay by the Agilent Seahorse XFe/XF96 analyzer (Agilent Technologies, Santa Clara, CA, USA). The mitochondrial respiration was measured during exposure to different substrates (glutamate, malate, succinate, and pyruvic acid). The final concentration of the injections was as follows: ADP was 1 mM, oligomycin was 2.5 mg/ml, FCCP was 2.2 mM/ml and rotenone was 2 mM/ml [19].

### Statistics

Data analyses were performed with GraphPad Prism 8.0 and Image J [20]. CCM data were processed by mixOmics packages with R software [21]. A Partial Least Squared discriminant analysis (PLS-DA) was performed to visualize general clustering and trends. PLS-DA scored plots of components one and two, comparing different diet groups were generated. KEGG pathway analysis was conducted in MetaboAnalyst 4.0. For non-CCM data, two group comparisons were performed using a paired two-tailed Student’s *t* test. The data of more than two groups were analyzed using one-way ANOVA followed by Turkey’s post-hoc *t* tests to compare all possible pairs of experimental groups. A value of *P* < 0.05 was considered significant.

## Results

### Loss of PEX16 leads to disappearance of peroxisomal membrane and hepatic steatosis

**Fig. 1:**
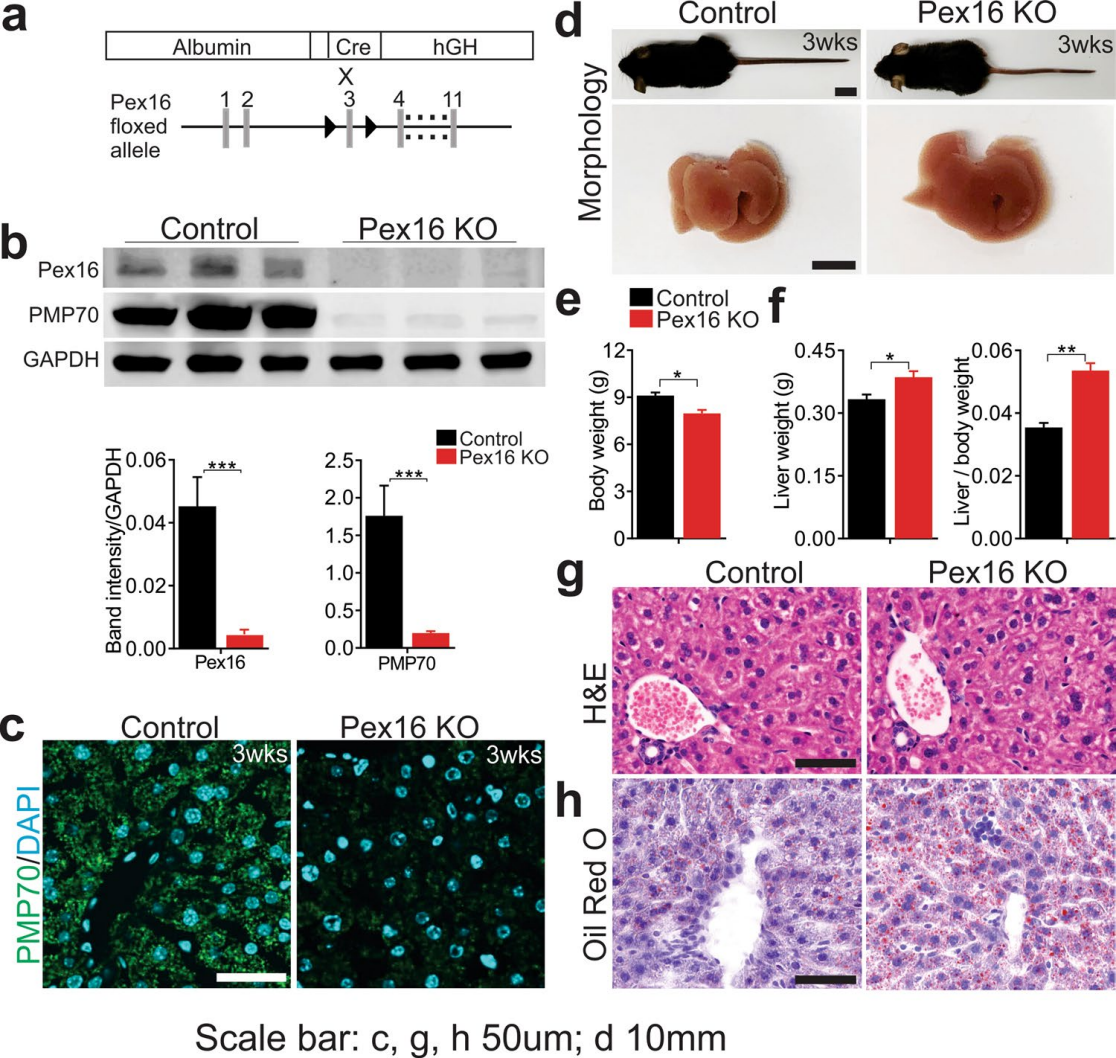
Hepatic steatosis in 3-week weanlings with hepatocyte specific deletion of *Pex16*. **a** Schematic of *Albumin-Cre*-mediated *Pex16* gene deletion. **b** Immunoblotting of PEX16 and PMP70 in wild-type and *Pex16* KO liver, and quantification of band intensity relative to GAPDH. **c** Immunofluorescence of PMP70 in control and *Pex16* KO liver. **d** Mouse and liver morphology of wild-type and *Pex16* KO groups. **e** Quantification of body weight and **f** Liver weight and liver to body weight ratio of control mice and *Pex16* KO mice. **g** H&E staining and **h** Oil red O staining of wild-type and *Pex16* KO liver. Data are represented as mean ± SEM, *n* = 6 mice per group. Scale bar: **c**, **g**, **h** 50 µm, **d** 10 mm. *p < 0.05, **p < 0.01, ***p < 0.001. See also Fig. S1

We generated a liver specific *Pex16* knockout (*Pex16* KO) mouse model by Albumin-Cre-mediated homologous recombination of a floxed allele (Fig. 1a). Western blot analysis of liver tissue showed the loss of PEX16 and peroxisomal membrane protein 70 (PMP70) at postnatal week 3, indicating efficient *Pex*16 deletion and peroxisome loss (Fig. 1b). Decreased immunofluorescence of PMP70 confirmed peroxisome ablation in hepatocytes (Fig. 1c, Fig. S1a). *Pex16* KO mice had a decreased body weight compared to wild-type control animals at postnatal week 3 (Fig. 1d, e). However, the liver weight adjusted for the body weight of *Pex16* KO mice was significantly increased (Fig. 1d, f). Although the structure and morphology of their liver tissue appeared normal (Fig. 1g), the Oil Red O staining revealed an increased presence of oil droplets in the livers of *Pex16* KO mice at 3 weeks (Fig. 1h) and 5 weeks of age (Fig. S1b). Together, these results show that hepatic loss of PEX16 leads to the disappearance of peroxisomes and hepatic steatosis.

### Nutritional stress induced by a low protein diet exacerbates hepatic steatosis caused by the loss of peroxisomes

**Fig. 2:**
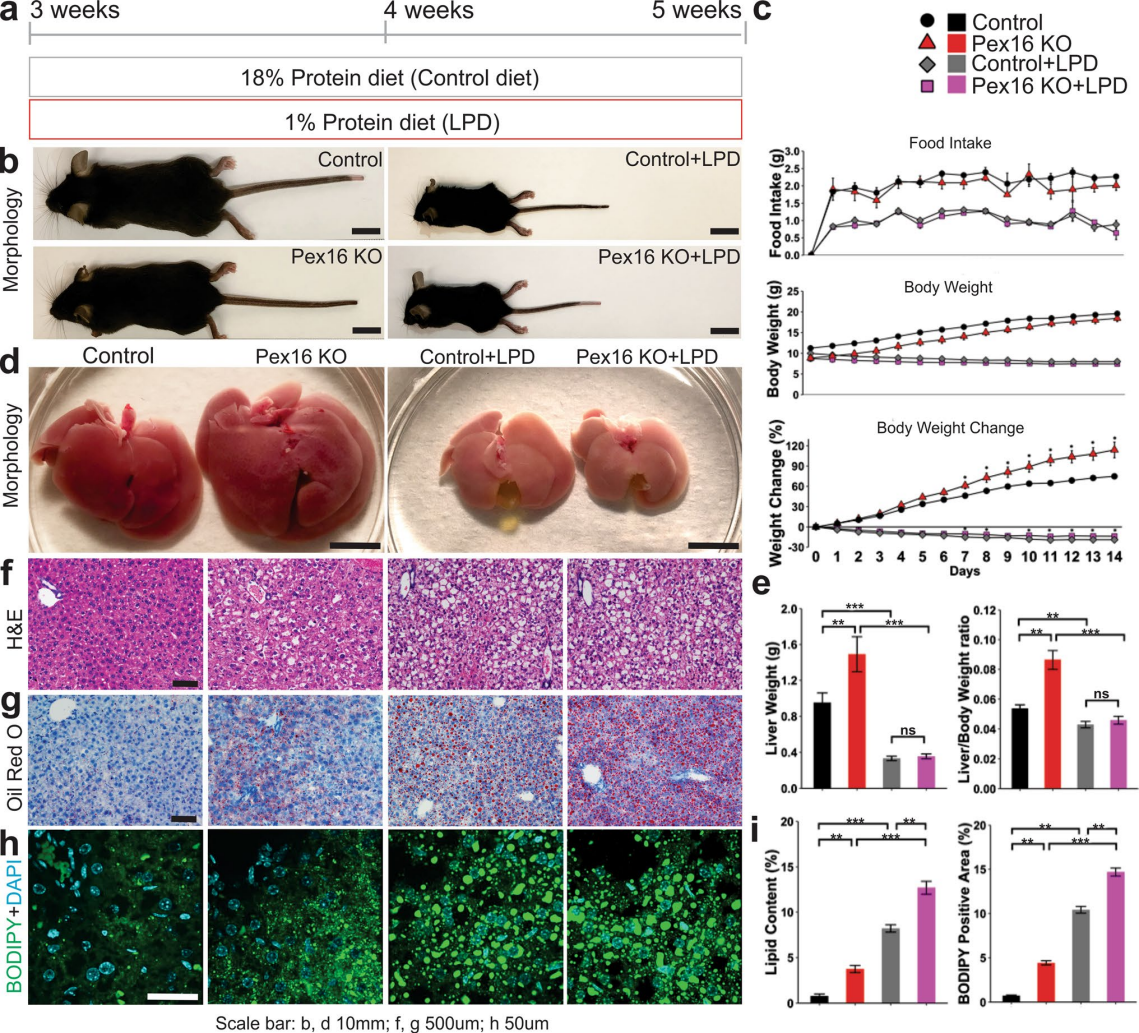
Growth delay and severe hepatic steatosis in LPD fed *Pex16* KO mice. **a** Schematic of experimental design. **b** Mouse morphology of wild-type and *Pex16* KO mice fed a control diet or LPD. **c** Quantification of food intake, body weight and body weight change. **d** Liver morphology of mice after 2 weeks of control diet or LPD. **e** Quantification of liver weight and liver to body weight ratio. **f** Liver H&E staining of wild-type and *Pex16* KO mice fed a control diet or LPD. **g** Oil red O staining. **h** Immunofluorescence of BODIPY staining. **i** Quantification of lipid droplet content from Oil Red O and BODIPY staining. Data are represented as mean ± SEM, *n* = 6–8 mice per group. Scale bar: **b**, **d** 10 mm, **f**, **g** 500 µm, **h** 50 µm. *p < 0.05, **p < 0.01, ***p < 0.001. See also Fig. S2

To determine susceptibility to nutritional stress, we subjected *Pex16* KO mice to a low protein diet (LPD) (Fig. S2) for 2 weeks starting at weaning (age 3 weeks) (Fig. 2a). As expected, body weight significantly decreased during LPD feeding in both *Pex16* KO mice and wild-type controls, whereas caloric intake was similar when corrected for body weight (Fig. 2b, c). The size and weight of the liver relative to body weight also decreased in mice fed an LPD (Fig. 2d, e). However, the liver enlargement seen in the *Pex16* KO mice was no longer observed when these animals were fed LPD (Figs. 1d, f, 2d, e). Histological analysis showed more vacuoles in the livers of *Pex16* KO mice, an effect exacerbated by the LPD (Fig. 2f). Oil Red O staining confirmed that these vacuoles corresponded to lipid droplets (Fig. 2g). Lipid droplets were fewer and smaller in the *Pex16* KO mice fed a regular diet compared to the wild-type mice fed an LPD (Fig. 2g, h). In contrast, there were more lipid droplets in the LPD-fed *Pex16* KOs compared to the wild-type controls fed the LPD. Lipid staining with BODIPY further demonstrated that the *Pex16* KOs fed LPD had the most severe hepatic steatosis (Fig. 2h, i).

### Loss of peroxisomes leads to disturbed hepatic energy homeostasis, which is exacerbated by nutritional stress

**Fig. 3:**
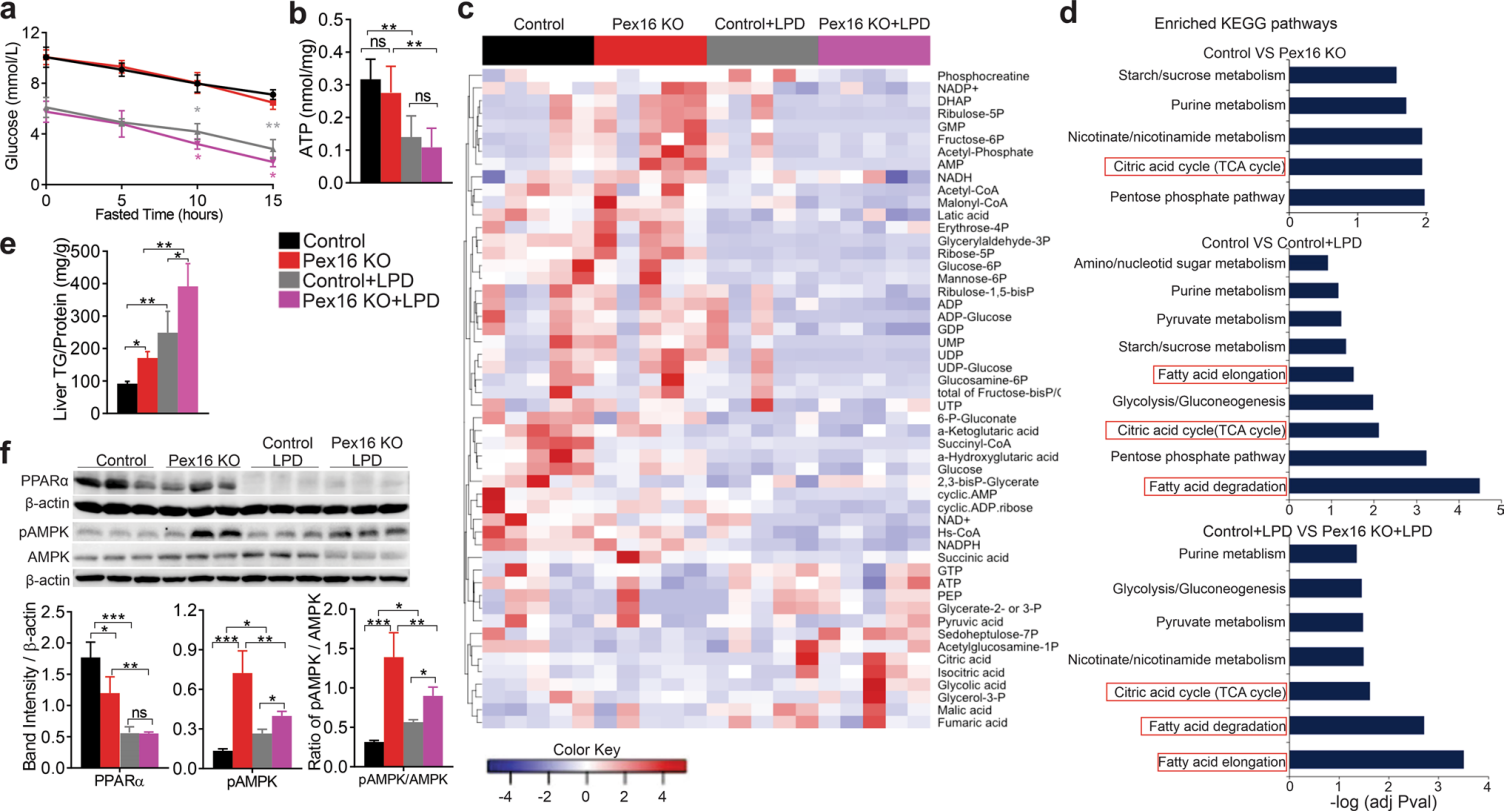
Loss of *Pex16* leads to changes in hepatic central carbon metabolism (CCM) exacerbated by LPD feeding in mice. **a** Blood glucose test in mice fasted for 15 h. Grey asterisks indicate significance between wild-type fed either control or LPD diets, pink asterisks indicate significance between wild-type fed LPD vs *Pex16* KO fed LPD. **b** Hepatic ATP levels. **c** Heat map presenting cluster analysis of CCM data. High level is in red, and low level is in blue as per legend. **d** KEGG pathways enriched for hepatic metabolism related pathways. The most affected pathways are related to citric acid cycle (TCA cycle) and fatty acid degradation/elongation (highlighted in red). **e** Hepatic triglycerides (TG) assay. **f** Western blots of hepatic PPARα, AMPKα and p-AMPKα, and quantification of band intensity relative to β-actin and ratio of p-AMPKα/AMPKα. Data are represented as mean ± SEM, *n* = 6–8 mice per group. *p < 0.05, **p < 0.01, ***p < 0.001. See also Fig. S3

To determine the effect of peroxisome depletion on liver metabolism during normal feeding and nutritional stress, we measured fasting blood glucose levels in *Pex16* KOs. During fasting, the liver becomes primarily responsible for gluconeogenesis, a process that consumes ATP in the liver. Fasting glucose was not affected in the *Pex16* KOs compared to the wild-type mice (Fig. 3a, Fig. S3a). However, it was reduced by LPD and exacerbated by the loss of PEX16, indicating reduced hepatic glucose production. Similarly, ATP levels decreased after LPD feeding in *Pex16* KO and wild-type mice (Fig. 3b). We further analyzed the hepatic intermediate metabolome targeting the central carbon metabolism. A total of 53 metabolites were detected, and *Pex16* KOs showed a distinct metabolic profile from wild-type controls (Fig. S3b). With LPD feeding, these differences between *Pex16* KOs and wild-type controls were still apparent, but the overall differences in metabolite profile appeared to be linked to the diet. Levels of each metabolite are shown in the heat map and the tables (Fig. 3c, and Tables S4, S5). Pathway enrichment analysis revealed that the TCA cycle was affected by both the loss of PEX16 and by LPD feeding (Fig. 3d). Specifically, entry into the TCA cycle appeared to be blocked in wild-type mice fed an LPD as shown by an increase in citrate and a concomitant decrease in acetyl-CoA. This change was more pronounced in the *Pex16* KOs fed an LPD associated with a substantial reduction in NAD^+^ content. These results indicate that loss of PEX16 affects overall energy homeostasis and induces a metabolic stress pattern that shows some similarities to that induced by LPD feeding.

### *Pex16* KO mice show increased triglyceride content despite the increase in the expression of β-oxidation genes

To better understand the etiology of hepatic steatosis caused by the loss of PEX16, we assessed relevant lipid pathways in the liver. We found that the loss of peroxisomes led to increased hepatic triglycerides, which was exacerbated by an LPD (Fig. 3e). This observation was consistent with our histological findings. As hepatic steatosis can be caused by increased lipogenesis, we examined the expression levels of lipogenesis regulatory genes. qPCR of acetyl-CoA carboxylase alpha (*Acaca*), considered a rate-limiting enzyme for lipogenesis, and fatty acid synthase (*Fas*) were decreased in the *Pex16* KO mice, as well as in both the wild-type and *Pex16* KO mice that were fed an LPD (Fig. S3c). We then evaluated the inhibition of the lipogenic pathway by assessing the phosphorylation status of AMP-activated protein kinase alpha (AMPKα) in the liver. Phosphorylated AMPKα protein levels and pAMPKα/ AMPkα were increased by both loss of PEX16 and LPD feeding, but the effect of diet was more modest (Fig. 3f). Consistent with increased AMPKα phosphorylation, the expression of genes regulating mitochondrial β-oxidation, peroxisomal β-oxidation and CoA formation were increased in *Pex16* KO mice fed a control diet (Fig. S3d–f). However, these increases in β-oxidation genes did not occur in *Pex16* KO mice fed an LPD. Instead, both wild-type mice and *Pex16* KO mice on an LPD showed a significant decrease in the expression of β-oxidation genes. The protein level of nuclear hormone receptor peroxisome proliferator-activated receptor alpha (PPARα), an important transcriptional regulator of β-oxidation, was reduced in the *Pex16* KO animals, and it was greatly decreased by LPD feeding in both strains (Fig. 3f). This result suggests that the increase in steatosis in the LPD mice may be caused by a decrease in β-oxidation. However, for the *Pex16* KO mice in control diet conditions, the increase in triglyceride levels and lipid droplets was observed in their livers despite an increase in β-oxidation gene expression, suggesting a potentially altered mitochondrial function.

### Hepatic mitochondrial dynamics are differentially altered in response to loss of peroxisomes and nutritional stress

**Fig. 4:**
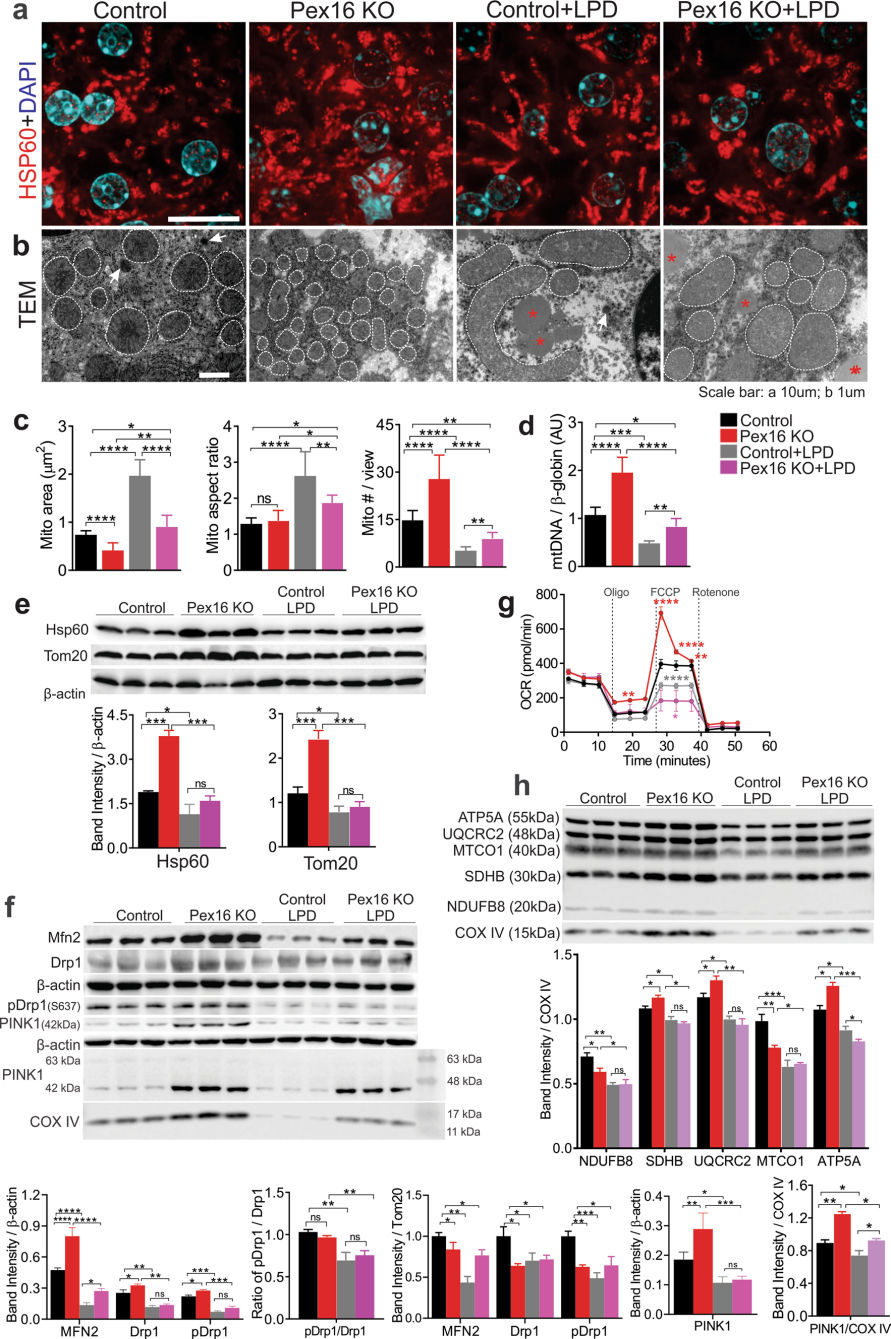
Loss of *Pex16* and LPD feeding differentially affect mitochondrial stress. **a** Immunofluorescence of HSP60 in hepatocytes of liver sections. **b** Transmission electron microscopy (TEM) images (× 20,000), hepatocyte mitochondria are outlined in white, arrows indicate hepatocyte peroxisomes, asterisks indicate fat granules. **c** Quantification of mitochondrial area, mitochondrial aspect ratio (length/width) and mitochondrial number from TEM images. **d** qPCR analysis of hepatocyte mitochondrial number. **e** Western blots of hepatic mitochondrial markers HSP60 and TOM20, and quantification of band intensity relative to β-actin. **f** Western blots of hepatic MFN2, DRP1, pDRP1, and PINK1 of liver tissue and isolated mitochondria, quantification of band intensity relative to β-actin, TOM20 and COX IV respectively and the ratio of pDRP1/DRP1. **g** Hepatocyte mitochondrial respiration/function was determined by oxygen consumption rate (OCR). Red asterisks indicate significance between wild-type vs *Pex16*KO, grey asterisks indicate significance between wild-type vs wild-type fed an LPD, pink asterisks indicate significance between wild-type + LPD vs *Pex16* KO + LPD liver. **h** Western blots of mitochondrial electron transport chain (ETC) complexes subunits, NDUFB8 (Complex I), SDHB (Complex II), UQCRC2 (Complex III), MTCO1 (Complex IV) and ATP5A (Complex V) from isolated hepatic mitochondria, quantification of band intensity relative to COX IV. Data are represented as mean ± SEM, *n* = 6–8 mice per group. Scale bar: **a** 10 µm and **b** 1 µm. *p < 0.05, **p < 0.01, ***p < 0.001, ****p < 0.0001. See also Fig. S4

Mitochondria are dynamic organelles. They elongate and divide to meet the metabolic needs of the cells, thus their morphology reflects the bioenergetic status of the cell [8]. For instance, acute nutrient deprivation leads to mitochondria elongation, which promotes increased oxidative phosphorylation and ATP production, and simultaneously prevents non-specific bulk degradation of mitochondria [22–24]. Conversely, extensive mitochondrial fission occurs during oxidative stress caused by oxygen deprivation or chemical inhibition of the mitochondrial respiration change complexes [25]. To assess the mechanism of the disruption of hepatic energy homeostasis in the *Pex16* KO mice, we examined their mitochondria in normal conditions and during metabolic stress. Immunofluorescent microscopy imaging of hepatocytes stained for the mitochondrial heat shock protein 60 (HSP60) (Fig. 4a) and transmission electron microscopy (Fig. 4b, Fig. S4a) were performed to compare mitochondrial morphology between the different mouse strains and conditions.

In comparison to the wild-type mice, there were more but smaller mitochondria in the *Pex16* KO mice hepatocytes in both metabolic conditions (Fig. 4a, b, circles with dashed lines). Quantification of the mitochondria size (area) and number verified their differences (Fig. 4c). The increase in mitochondria in the *Pex16 KO* animals was further corroborated by both an increase in the mitochondrial DNA (mtDNA) copy number (Fig. 4d) and by higher levels of the outer membrane translocase 20 (TOM20) and HSP60 (Fig. 4e) compared to wild-type controls.

Under LPD-induced metabolic stress, both wild-type and *Pex16* KO mice had larger but fewer hepatic mitochondria (Fig. 4a–c), as assessed by protein levels and mtDNA. This effect was the strongest in the wild-type mice, as *Pex16* KO mice fed an LPD had smaller and more numerous mitochondria than the wild-type mice fed an LPD (Fig. 4a–e). We also assessed levels of hepatic peroxisomal proteins and found that wild types fed an LPD had substantially decreased levels of PMP70 and PEX16, which reflected the results shown in livers of amino acid starved rats [17] (Fig. S4b).

We next assessed whether changes in mitochondrial morphometrics were related to mitochondrial fusion and fission by assessing the level of MFN2 and DRP1 respectively. We showed by western blot that MFN2, total DRP1, and phospho-DRP1-S637 (pDRP1) were increased in the *Pex16* KO mice compared to the wild types (Fig. 4f). However, as mitochondria are more numerous in the hepatocytes of *Pex16* KO mice (Fig. 4a–d), we normalized MFN2 and DRP1 expression to that of TOM20 and found that these proteins were lower in mitochondria of *Pex16* KO compared to those in the wild-type animals. However, the pDRP1/DRP1 ratio did not differ between strains fed LPD. The dynamics of mitochondrial fission and fusion are complex, and our data are inconclusive regarding the link between increased fission and smaller mitochondrial size in *Pex16* KO hepatocytes. The complexity may derive from mitochondria incurring different or compounded damage in response to the loss of peroxisomes in normal versus LPD feeding, thus, the insults could induce a different combination of repair responses leading to an intermediate phenotype in mitochondrial morphology.

### Loss of peroxisomes exacerbates nutritional stress-induced mitochondrial dysfunction

To assess whether the mitochondrial morphological changes were associated with impaired function, we evaluated mitochondrial health by measuring the oxygen consumption rate (OCR) of isolated hepatic mitochondria. Their basal and maximal respiration was assessed in the different strains fed a normal diet or LPD. Baseline OCR did not differ between groups, but the *Pex16* KO mice fed a normal diet had a higher reserve respiration capacity (maximal respiration) compared to wild-type controls (Fig. 4g). Interestingly, the hepatic mitochondria of the *Pex16* KO mice showed a gradual decrease in OCR following FCCP treatment, which was not observed in wild-type mice. In both LPD-fed groups, mitochondria showed very little respiration reserve as their maximal respiration did not significantly increase over their basal OCR. These results reveal that hepatic mitochondrial function is not impaired by loss of peroxisomes, which is consistent with the unchanged levels of ATP at basal conditions. Instead, the higher reserve respiration capacity in the peroxisome-deficient mice suggests that their mitochondria may have adapted to the changed cellular condition and resulted in mitochondria with a higher capacity to produce ATP. However, under nutritional stress, the mitochondria of *Pex16* KO animals have lost their increased reserve respiration capacity.

Next, we assessed the complexes of the electron transport chain (ETC) in isolated mitochondria. We normalized mitochondria complex proteins detected with immunoblotting to cytochrome *c* oxidase subunit 4 (COX IV), the terminal enzyme in the respiratory chain to allow comparison of relative changes in these complex proteins as described by others [26]. When normalized to COX IV, mitochondrial complex II (SDHB), III (UQC RC2) and V (ATP5A) were modestly increased in the mitochondria isolated from *Pex16* KOs compared to wild types, while complex I (NDUFB8) and IV (MTCO1) were modestly decreased (Fig. 4h). LPD feeding decreased all the complexes compared to normal-fed animals, but no differences were observed between the two LPD-fed groups except for complex V. In addition, the normalization protein COX IV differed across conditions where levels appeared to increase in the mitochondria of *Pex16* KO mice, which reflects an increase in mitochondria mass observed in these mice compared to the wild-type mice (Fig. 4c). Together with the mitochondria stress test, these results suggest that the *Pex16* KO animals are able to adapt to peroxisomal loss under normal conditions by increasing mitochondria numbers and the reserve capacity of the mitochondria. However, the increased reserve capacity is lost under metabolic stress.

### PEX16 deficiency alters mitochondria quality control related to a decrease in autophagy

In both normal and LPD-fed conditions, the mitochondria of the *Pex16 KO* animals were more fragmented and more numerous but showed no difference in ATP production or basal respiration compared to their wild-type counterpart. As these observations could suggest altered mitochondrial quality control, we next examined for defects in mitophagy, the autophagic degradation of mitochondria [9]. An indicator of mitochondria damage is the accumulation of PTEN-induced kinase 1 (PINK1). PINK1 is targeted to the mitochondria and is readily cleaved upon entry by the inner membrane protease PARL, the cleaved products are retro-translocated and degraded by proteasomes in healthy mitochondria, whereas full-length PINK1 accumulates in damaged mitochondria [27]. This accumulation initiates PARKIN-dependent mitophagy, which induces mitochondria fragmentation and the recruitment of autophagic factors to the damaged mitochondria [28]. Therefore, we first assessed whether PINK1 accumulated in *Pex16* KO livers. Full-length PINK1 was barely detectable in all conditions (Fig. 4f). When normalized to β-actin, we found that, in wild-type mice, LPD feeding led to a small but significant decrease in PINK1 compared to normal diet feeding. However, we saw an increase in cleaved PINK1 (42 kDa fragment) in the *Pex16* KO mice fed a control diet or LPD (Fig. 4f). Similar results were observed when cleaved PINK1 was measured in isolated mitochondria and adjusted for COX IV (Fig. 4f). The significance of the accumulation of cleaved PINK1 is unclear, but it is known that in healthy mitochondria, cleaved PINK1 is efficiently removed from the mitochondria by retro-translocation and degradation [29, 30]. However, we did not observe an accumulation of full-length PINK1, which would be indicative of significant mitochondrial damage, typically observed during chemically induced mitochondria damage [31].

**Fig. 5:**
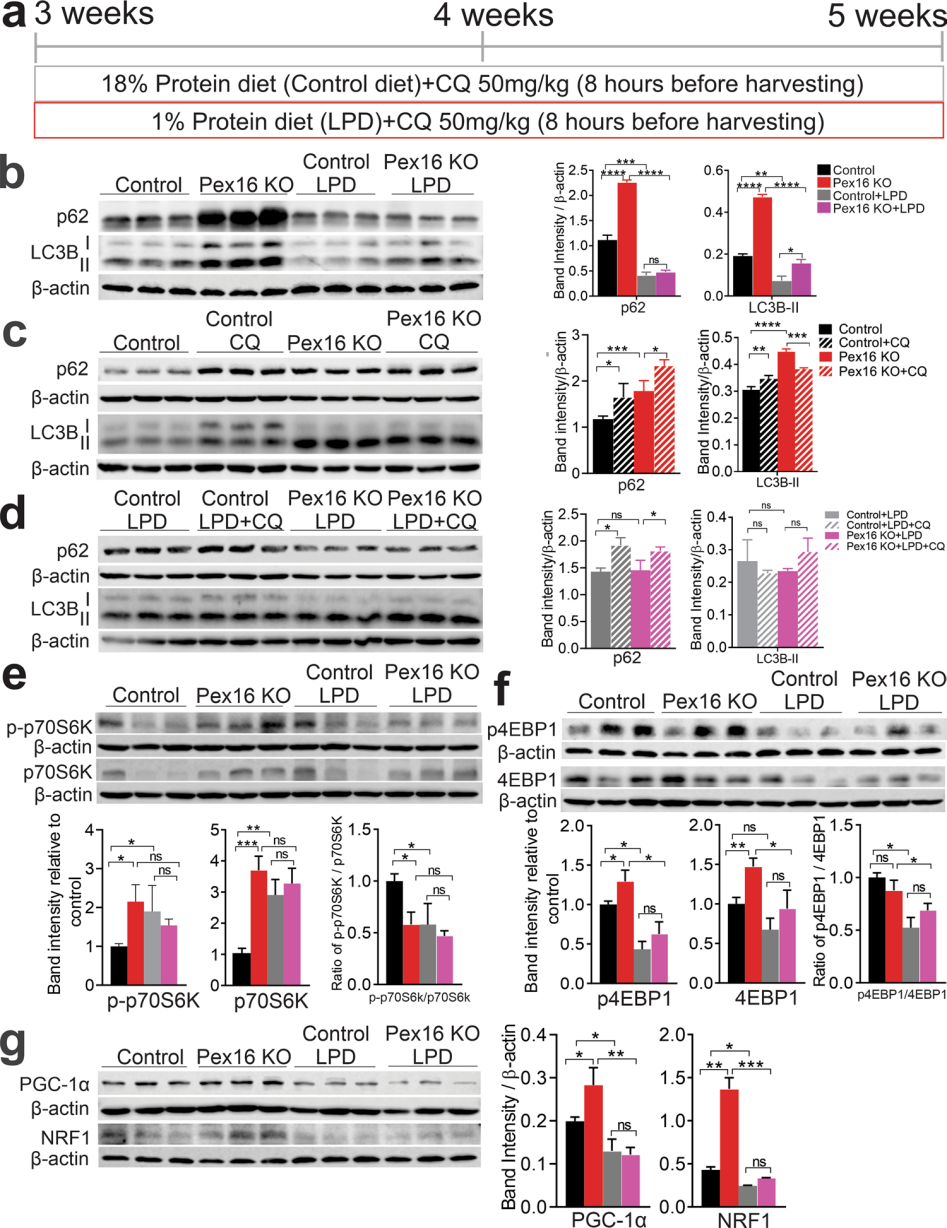
Loss of *Pex16* and LPD feeding differentially affect autophagy and mitochondrial biogenesis. **a** Schematic of mice treated with chloroquine (CQ) for 8 h under wild-type control and *Pex*16 KO mice fed a control diet or LPD. **b** Western blots of hepatic p62, LC3B-I and II, and quantification of band intensity relative to β-actin of wild-type and *Pex16* KO mice fed a control diet or LPD. **c** Western blots of hepatic p62 and LC3B-I and II, and quantification of band intensity relative to β-actin of wild-type and *Pex16* KO mice fed a control diet and treated by CQ. **d** Western blots of hepatic p62 and LC3B-I and II, and quantification of band intensity relative to β-actin of wild-type and *Pex16* KO mice fed an LPD diet and treated by CQ. **e** Western blot of mTOR downstream effector p-p70S6K and p70S6K, and quantification of band intensity relative to β-actin and the ratio of p-p70S6K/p70S6K. **f** Western blot of mTOR downstream effector p4EBP1, 4EBP1, and quantification of band intensity relative to β-actin and the ratio of p4EBP1/4EBP1. **g** Western blot of mitochondrial biogenesis marker PGC-1α and NRF1, and quantification of band intensity relative to β-actin. Data are represented as mean ± SEM, *n* = 6–8 mice per group. *p < 0.05, **p < 0.01, ***p < 0.001, ****p < 0.0001. *CQ* chloroquine. See also Fig. S5

We next examined whether autophagy was downregulated in *Pex16* KO livers. We first analyzed the autophagy receptor protein sequestosome-1 (p62) and microtubule-associated protein 1A/1B light chain 3B (LC3B). p62 is turned over by autophagy, thus it accumulates in cells when autophagy or autophagy flux is defective [32]. LC3B-I is the cleaved version of the proLC3B before it is conjugated to phosphatidylethanolamine. The LC3B-PE, also known as LC3B-II, has increased mobility on SDS-PAGE due to lipidation (Fig. 5b), and it can be used to measure the amount of autophagosomes in the cell. We found that both p62 and LC3B-II were higher in the *Pex16* KO mice on regular feed, whereas LC3B-II was observed to be higher in the LPD-fed *Pex16* KO mice compared to LPD-fed wild types (Fig. 5b). However, p62 and LC3B-II levels were lower in the LPD mice than the mice with regular feed, suggesting a difference in autophagy induction. Since a higher p62 level and LC3B-II can suggest a defect in autophagy flux (the rate of autophagy substrate degraded in lysosomes), we measured the LC3B-II in mice treated with the lysosomotropic agent chloroquine to inhibit lysosome degradation. Acute administration of chloroquine to inhibit autophagic flux led to a moderate increase in p62 levels for all conditions compared to the untreated animals as expected (Fig. 5c, d). The wild-type control-fed mice showed a significant increase in LC3B-II content after chloroquine treatment (Fig. 5c). However, the *Pex16* KO fed the control diet actually showed a modest decrease in their LC3B-II content upon chloroquine treatment compared to their untreated counterpart. The LPD groups showed no increase after chloroquine (Fig. 5c). These experiments suggest that the rate of autophagy flux is slowed in the livers of *Pex16* KO mice compared to wild-type animals, which may contribute to the accumulation of mitochondria in the liver of *Pex16* KO mice (Fig. 4a–e).mTORC1 is the main regulator of autophagy and the inhibition of mTORC1 kinase activity induces autophagy [33]. To further investigate whether the impact of functional loss of PEX16 on autophagy is related to changes in mTORC1 activation, we analyzed phosphorylated p70S6K (p-p70S6K) and phosphorylated 4EBP1 (p-4EBP1), two downstream substrates of mTORC1 kinase activity. p-p70S6K/p70S6K ratio was down-regulated in the *Pex16* KO mice consistent with inhibition of mTORC1, but p-4EBP1/4EBP1 ratio was not changed (Fig. 5e, f). Both strains fed LPD showed a decrease in both p-p70S6K/p70S6K and p-4EBP1/4EBP1 ratios supporting that there is an increase in autophagy. Interestingly, loss of functional peroxisomes increased total protein levels of 4EBP1 and p70S6K, with mRNA levels p70S6K most dramatically upregulated in *Pex16* KO mice (Fig. S5a, S5b). Increased ROS has been shown to upregulate 4EBP and S6K transcription [34] and could be a mechanism that explains the gene and protein expression changes in our model. While the increase in 4EBP1 and p70S6K levels in the *Pex16* KOs limits our ability to draw firm conclusions on mTORC1 activation, this effect may explain the lack of a difference in the p-4EBP1/4EBP1 ratio in comparison to wild types. In summary, the accumulation of altered mitochondria in the *Pex16* KO mice may result from a downstream impairment in overall autophagic flux, despite indications that mTORC1 is inhibited.

### *Pex16* KO mice show increased mitochondrial biogenesis

To determine whether the changes in mitochondrial content were related to mitochondrial biogenesis, we analyzed the levels of peroxisome-proliferator-activated receptor coactivator-1α (PGC-1α) and its downstream target nuclear respiratory factor 1 (NRF1) [35]. PGC-1α and NRF1 protein (Fig. 5g) and *Pgc-1α* and *Nrf1* mRNA (Fig. S5c, S5d) were elevated in the liver of *Pex16* KO compared to the wild types, consistent with the increase in mitochondrial number and mass. In contrast, PGC-1α content was decreased by LPD feeding to similar levels in the wild type mice and *Pex16* KOs to the normal feeding (Fig. 5g, Fig. S5c). Our results indicate that increased mitochondrial content in *Pex16* KO is related to both reduced autophagy and increased mitochondrial biogenesis. In contrast, the reduced mitochondrial content related to LPD feeding appears to be caused by reduced mitochondrial biogenesis.

### Activation of PPARα improves hepatic steatosis caused by loss of functional peroxisomes and low protein diet feeding

**Fig. 6:**
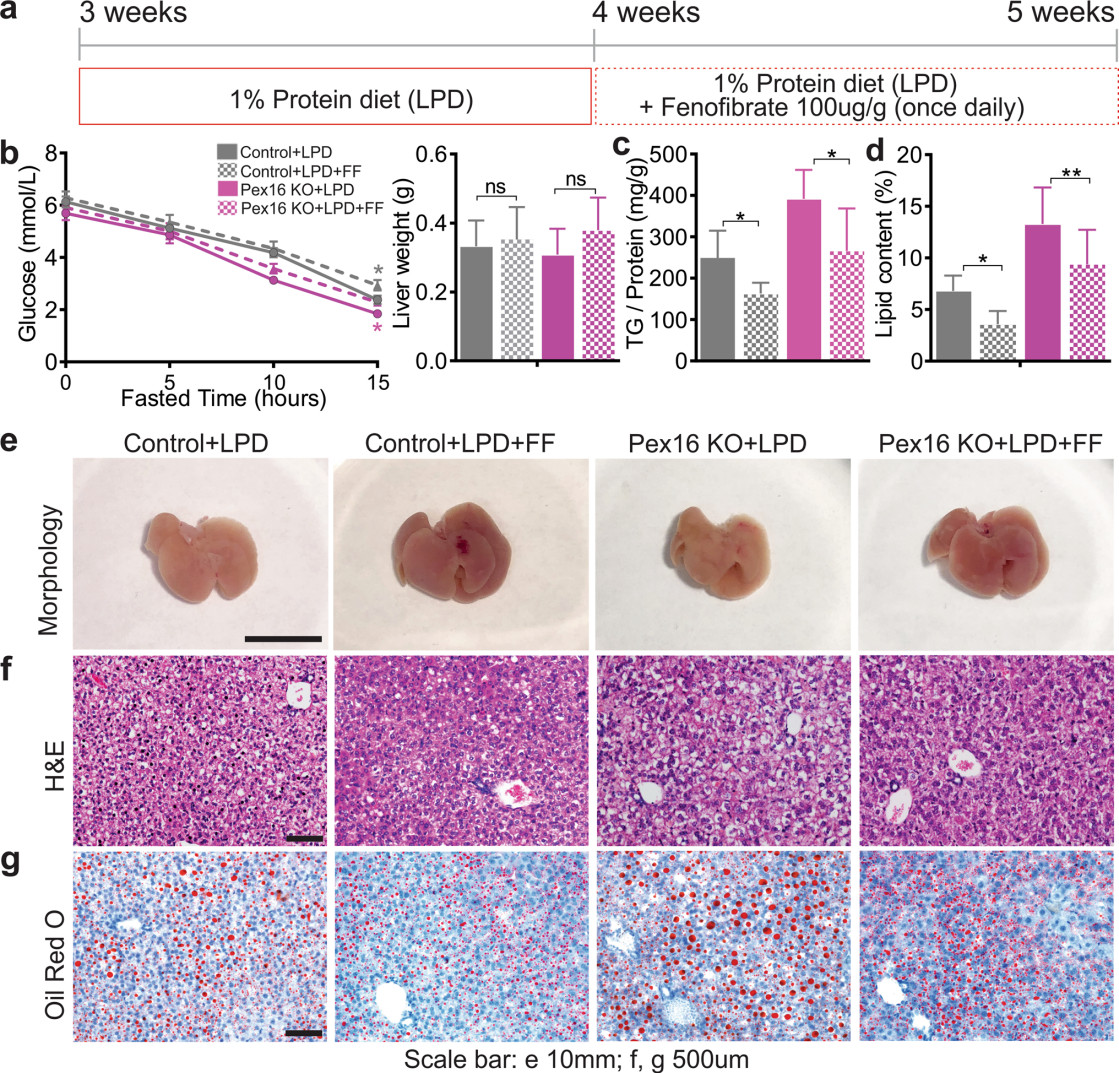
Fenofibrate (FF) treatment reduces hepatic steatosis in *Pex16* KO and wild-type mice fed an LPD. **a** Schematic of experiments where mice fed an LPD were treated with fenofibrate through gavage for 1 week. **b** Blood glucose test in mice fasted for 15 h, and liver weight. Grey asterisks indicate significance between wild-type + LPD vs wild-type + LPD + FF, pink asterisks indicate significance between *Pex16* KO + LPD vs *Pex16* KO + LPD + FF. **c** Hepatic triglycerides (TG) assay. **d** Quantification of lipids from Oil Red O staining sections shown in **g**. **e** Liver morphology of LPD + FF treated wild-type and *Pex16* KO mice. **f** Liver H&E staining. **g** Liver Oil red O staining. Data are represented as mean ± SEM, *n* = 6–8 mice per group. Scale bar: **e** 10 mm, **f**, **g** 500 µm. *p < 0.05, **p < 0.01. *FF* fenofibrate. See also Fig. S6

PPARα is a nuclear receptor that enhances mitochondrial biogenesis and β-oxidation, and it was recently found to induce autophagy [36]. Therefore, we next assessed whether PPARα activation with fenofibrate could improve hepatic metabolism in *Pex16* KO mice fed either a control diet or an LPD (Fig. 6a). Fenofibrate did not affect body weight, liver weight, food intake, or fasting glucose concentrations in *Pex16* KO or wild-type mice fed a control diet or an LPD (Fig. 6b, Fig. S6a, b, S6g, h). However, fenofibrate treatment reduced hepatic steatosis in *Pex16* KO mice fed either a control diet or an LPD (Fig. 6c–g, Fig. S6f–h). Fenofibrate had a similar effect in wild types fed LPD, whereas vehicle treatment did not improve hepatic lipid accumulation (Fig. S6c–f, S6h).

### PPARα activation improves hepatic mitochondrial function independently of functional peroxisomes

**Fig. 7:**
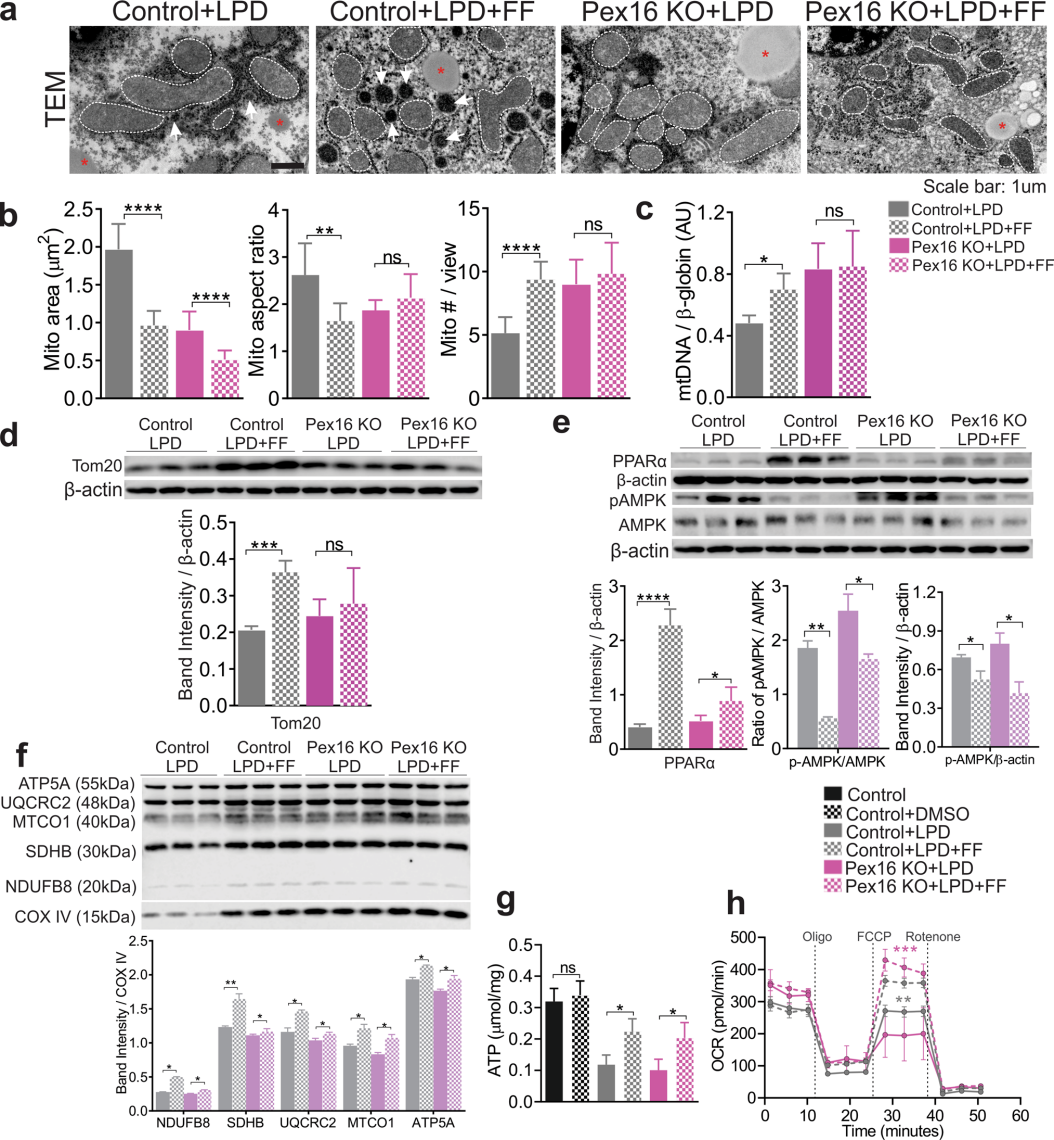
Fenofibrate (FF) treatment improves mitochondrial respiration in *Pex16* KO and wild-type mice fed an LPD. **a** Transmission electron microscopy (TEM) images (× 20,000), hepatocyte mitochondria are outlined in white, arrows indicate hepatocyte peroxisomes, asterisks indicate lipids. **b** Measurement of mitochondrial area, mitochondrial aspect ratio (length/width), and mitochondrial number from TEM images. **c** qPCR analysis of hepatocyte mitochondrial number. **d** Western blots of hepatic TOM20 and quantification of band intensity relative to β-actin. **e** Western blots of hepatic PPARα, p-AMPKα, AMPKα, quantification of band intensity relative to β-actin and the ratio of p-AMPKα/AMPKα. **f** Western blots of mitochondrial electron transport chain (ETC) complexes subunits, NDUFB8 (Complex I), SDHB (Complex II), UQCRC2 (Complex III), MTCO1 (Complex IV) and ATP5A (Complex V) from isolated hepatic mitochondria, quantification of band intensity relative to COX IV. **g** Hepatic ATP levels. **h** Hepatocyte mitochondrial respiration/function was determined by oxygen consumption rate (OCR). Grey asterisks indicate significance between wild-type + LPD vs wild-type + LPD + FF, pink asterisks indicate significance between *Pex16* KO + LPD vs *Pex16* KO + LPD + FF. Data are represented as mean ± SEM, *n* = 6–8 mice per group. Scale bar: **a** 1 µm, *p < 0.05, **p < 0.01, ***p < 0.001, ****p < 0.0001. *FF* fenofibrate. See also Fig. S7

We next examined the effect of activating PPARα on mitochondrial fitness in *Pex16* KOs fed a control diet or an LPD. Fenofibrate treatment increased peroxisome numbers in wild types fed LPD (Fig. 7a) consistent with an increase in PMP70 (Fig. S7e). Peroxisomal structures were not observed in the EM micrographs of the *Pex16* KO livers (Fig. 7a), although we did not perform alkaline 3,3′-diaminobenzidine staining to directly visualize peroxisomes. We therefore further validated our EM findings using immunofluorescence staining of PMP70, which showed an increase with fenofibrate treatment, including small increases in *Pex16* KO livers (Fig. S7e). However, this PMP70 staining co-localized with endothelial cells as indicated by co-staining with CD31, an endothelial marker (Fig. S7f). This indicates that fenofibrate treatment can stimulate non-hepatocyte peroxisome biogenesis. Mitochondria were smaller after fenofibrate treatment in *Pex16* KOs fed an LPD (Fig. 7a, b). However, their numbers were not changed (Fig. 7b). In contrast, the mitochondria of *Pex16* KOs on a control diet treated with fenofibrate showed no clear change in size but were reduced in number compared to untreated mice (Fig. S7a–c). In wild types fed LPD, fenofibrate treatment increased mitochondrial numbers, but these mitochondria were smaller and less elongated (Fig. 7a–c). Consistently, TOM20 protein levels and mtDNA were not significantly increased with fenofibrate treatment in the *Pex16* KOs fed an LPD but were increased in wild-type animals (Fig. 7c, d). These changes in mitochondria were associated with a fenofibrate-induced decrease in p-AMPKα and increase in PPARα protein levels (Fig. 7e). Also, mitochondrial content was not impacted by DMSO treatment in wild types fed a control diet (Fig. S7a–e).

As expected, fenofibrate treatment upregulated mRNA levels of most genes involved in mitochondrial β-oxidation, peroxisomal β-oxidation and CoA synthesis in *Pex16* KO and wild-type mice fed an LPD (Fig. S7g). Gene expression of lipogenesis markers *Acaca* and *Fasn* were downregulated in fenofibrate-treated mice but this response to treatment was absent in *Pex16* KO mice fed a control diet (Fig. S7h).

Mitochondrial protein complexes isolated from mitochondria were increased by fenofibrate in wild-type mice fed an LPD compared to untreated mice, although the effect appeared less pronounced in the *Pex16* KOs (Fig. 7f). No substantial effects of fenofibrate treatment on mitochondrial complex proteins were observed in the *Pex16* KOs fed a control diet, with a modest increase in complex IV and a small decrease in complex II and V (Fig. S7i). This data suggests that PPARα activation improves mitochondrial function in *Pex16* KOs fed an LPD through an increase in mitochondrial complex content without an overall effect on mitochondrial mass.

In line with these results, the ATP content was similarly increased in *Pex16* KOs fed an LPD after fenofibrate treatment compared to wild types (Fig. 7g). Finally, mitochondria showed improved respiration in the *Pex16* KOs with or without LPD feeding (Fig. 7h, Fig. S7j). These results indicate that PPARα activation improves mitochondrial fitness in mice subjected to LPD-induced nutritional stress despite an absence of functional peroxisomes.

### PPARα activation stimulates mitochondrial biogenesis and autophagy in *Pex16* KO mice

**Fig. 8:**
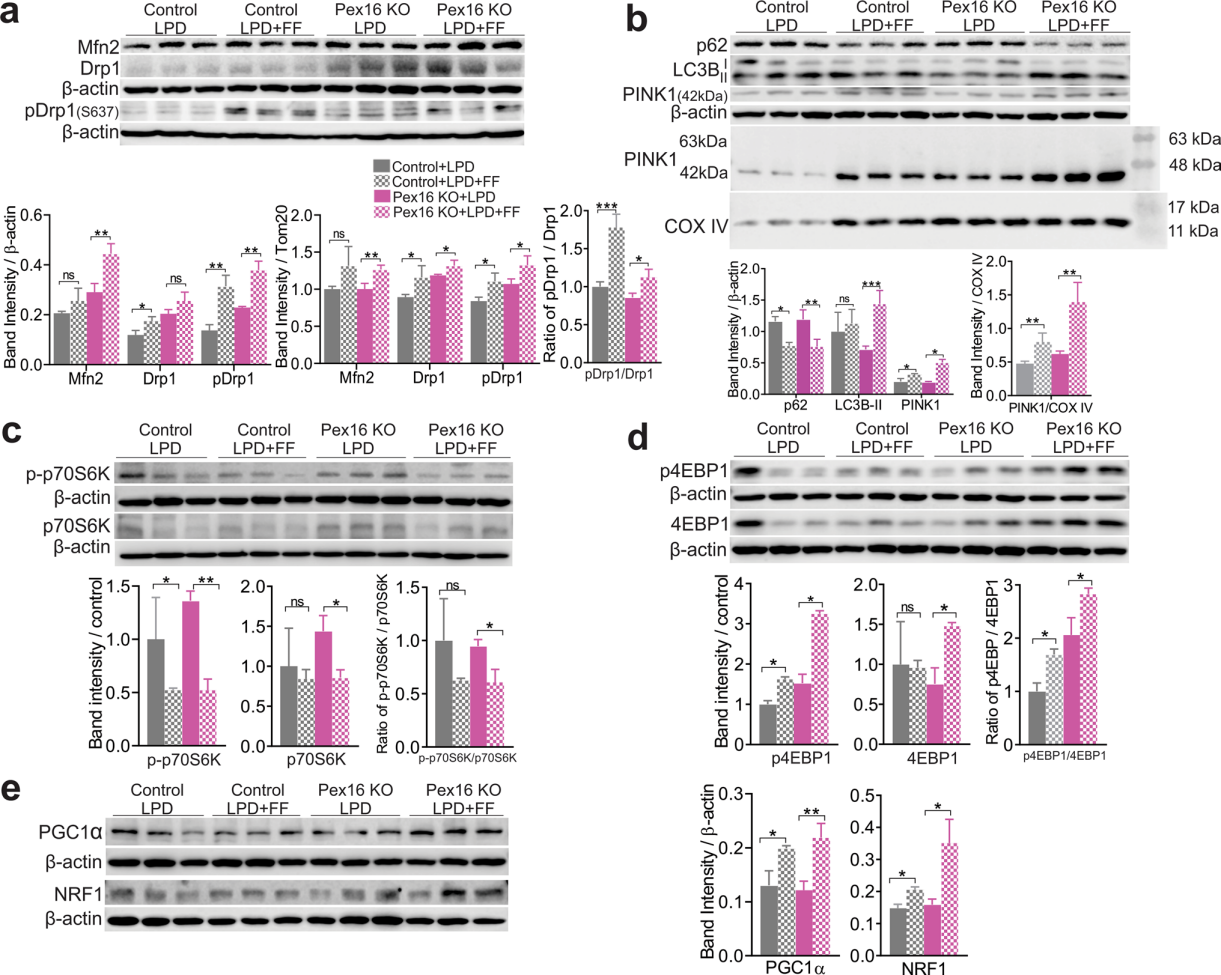
Fenofibrate (FF) treatment stimulates mitochondrial biogenesis and autophagy in *Pex16* KO and wild-type mice fed an LPD. **a** Western blots of hepatic MFN2, DR1, pDRP1, and quantification of band intensity relative to β-actin and TOM20, and the ratio of pDRP1/DRP1. **b** Western blot of hepatic autophagy and mitophagy markers p62, LC3B and PINK1 in liver lysates, and PINK1 in isolated hepatic mitochondria, quantification of band intensity relative to β-actin and COX IV. **c** Western blot of autophagy marker p-p70S6K and p70S6K, and quantification of band intensity relative to β-actin and the ratio of p-p70S6K/p70S6K. **d** Western blot of autophagy marker p4EBP1, 4EBP1, and quantification of band intensity relative to β-actin and the ratio of p4EBP1/4EBP1. **e** Western blot of mitochondrial biogenesis marker PGC-1α and NRF1, and quantification of band intensity relative to β-actin. Data are represented as mean ± SEM, *n* = 6–8 mice per group. *p < 0.05, **p < 0.01, ***p < 0.001. *FF* fenofibrate. See also Fig. S8

We next assessed the effects of PPARα activation on markers of mitochondrial dynamics, biogenesis, and autophagy. Both MFN2 protein level and pDRP1/DRP1 ratio were increased in the fenofibrate-treated *Pex16* KOs fed a control diet or an LPD compared to untreated animals (Fig. 8a, Fig. S8a). Both *Pex16* KOs and the wild-type animals fed an LPD and treated with fenofibrate showed an increase in autophagosome formation (ratio LC3B-II/I) and a decrease in p62 (Fig. 8b), which suggests an increase in autophagic flux. Similar changes in the autophagic factors were also observed in fenofibrate-treated *Pex16* KO mice fed the control diet (Fig. S8b).

The effects on autophagy were associated with a decrease in p-p70S6K/p70S6K indicating inhibition of mTOR (Fig. 8c). However, fenofibrate treatment increased p-4EBP1/4EBP1 in all mouse groups (Fig. 8d). mRNA levels of these factors showed a similar pattern (Fig. S8d). PGC-1α and NRF1 were upregulated in *Pex16* KOs as well as wild-type animals after fenofibrate treatment (Fig. 8e, Fig. S8e). Together, these data suggest that the improved mitochondrial health observed after PPARα activation in *Pex16* KOs fed an LPD is related to increased mitochondrial biogenesis and enhanced autophagy.

## Discussion

In this study, we describe the metabolic adaptation that occurs in a peroxisome-deficient hepatocytes model. The knockout of the peroxisomal biogenesis gene PEX16, which results in the loss of peroxisomal membrane, causes changes in mitochondria morphology and function. However, the hepatocytes of the *Pex16 KO* mice still produced ATP at levels comparable to wild-type animals (Fig. 3e), suggesting the observed mitochondria changes may act as a compensatory response in livers with impaired peroxisomal function. Functionally, mitochondria from *Pex16* KO hepatocytes have increased reserve respiration capacity. Further, the hepatocytes showed upregulation in mitochondrial biogenesis and reduction in autophagy. However, *Pex16* KO mice were less able to adapt to metabolic stress induced through LPD feeding. However, activating PPARα improved mitochondrial and overall metabolic function in the absence of functional peroxisomes in hepatocytes by stimulating mitochondrial biogenesis and autophagy. Our work not only provides further support to the importance of peroxisomes in maintaining mitochondria health, but it also reveals a certain adaptive capacity of hepatocytes to maintain mitochondrial function without functional peroxisomes.

### Loss of PEX16 and peroxisomes affects mitochondrial homeostasis and function

PEX16 is essential for peroxisome biogenesis as its absence results in the loss of peroxisomal membrane [37] and is associated with Zellweger syndrome [7] and leukodystrophy [38]. We developed a liver-specific *Pex16* KO mouse model as demonstrated by the loss of PEX16 and the peroxisome membrane protein, PMP70. These mice developed hepatic steatosis similar to other models of loss of peroxisome function, such as in PEX2 and PEX5 deficiency [12–14]. Mitochondrial abnormalities are found in patients with Zellweger syndrome [39] and mouse models with deletion of *Pex2* and *Pex5* [13, 14, 40], underscoring the role of peroxisomes in mitochondrial health. In this study, we showed that loss of *Pex16* leads to the accumulation of small mitochondria, but with only minor functional disruption (Fig. 3). Mitochondria morphological changes without ATP disruption were also reported in *Pex5* KO mice at 10 weeks of age [13] compared to our mice sacrificed at 5 weeks of age. The more severe hepatic mitochondrial changes found early in life were in whole body *Pex2* and *Pex5* KO models. It should be noted that these mice have very different peroxisome content. The *Pex16* KO livers are absent of any peroxisomal structures owing to the role of PEX16 in peroxisome membrane formation. At the same time, the KO of *Pex5* in hepatocytes leads to fewer but larger peroxisomal structures than in wild-type hepatocytes. The peroxisomal membrane proteins may have functions that affect mitochondria. Finally, the more severe phenotype in the whole body *Pex2* and *Pex5* KO models could have been related to extrahepatic pathophysiological effects secondarily affecting the liver [12, 14].

Interestingly, the stress test of mitochondria isolated from the *Pex16* KO showed a rapid loss of oxygen consumption rate upon treatment with FCCP. This decrease is unlikely due to a limitation of FCCP as the concentration of mitochondria used was the same as the wild-type mitochondria, which did not show this loss in its OCR upon FCCP treatment (Fig. 4g and Fig S7j). However, we cannot rule out a possible issue with substrate concentration. Further, we observed an accumulation in cleaved PINK1 in the *Pex16* KO liver, but the cause of this accumulation is not known. Given that PINK1 is readily cleaved by the inner membrane protease PARL, and retro-translocated out to the cytosol for degradation by the proteasome [31], an accumulation of the cleaved form of PINK1 may indicate a mild defect in the mitochondria. Finally, we also found that the *Pex16* KO mice fed an LPD were more susceptible to mitochondrial damage compared to the wild-type mice. Therefore, we propose that the *Pex16* KO mitochondria are more susceptible to metabolic stress-induced damage.

Peroxisomes have been proposed to act in regulating mitochondria redox homeostasis [2]. Smaller or fragmented mitochondria have decreased potential [8]. The smaller mitochondria observed in the *Pex16* KO mice are likely a compensation mechanism to prevent mitochondria-induced oxidative stress in the absence of peroxisomes. In fact, similar fragmentation in mitochondria is observed during hypoxia where DRP1 is activated to promote mitochondria fragmentation [41]. However, since a decreased potential is correlated with decreased ATP production, the *Pex16* KO mice appear to compensate by increasing the amount of mitochondria (Fig. 4). Finally, we observed that the *Pex16* KO mice have a higher maximum respiration capacity compared to wild-type mice and higher levels of ETC proteins, yet they do not produce more ATP. This suggests the *Pex16* KO hepatocyte mitochondria function is downregulated to minimize the production of ROS. Therefore, we propose that hepatocyte mitochondrial function in the *Pex16* KO mice may be maintained through a compensatory response. However, these changes make their mitochondria more susceptible to damage during metabolic stress.

The loss of PEX16 did lead to mild hepatic steatosis, even on a control diet, despite having preserved mitochondrial function. Hepatic steatosis can be caused by increased hepatic lipogenesis or extrahepatic lipolysis, with the latter leading to a higher flux of fatty acids to the liver. Alternatively, decreased β-oxidation or very-low-density lipoprotein secretion could contribute to hepatic lipid accumulation. Lipogenesis was unlikely to be responsible, given the reduced expression of lipogenic genes in the *Pex16* KO compared to wild-type mice. Increased lipolysis through enhanced lipoprotein or hormone-sensitive lipase activity was also less likely, given that this was a liver-specific KO model. Limited data suggest that very-low-density lipoprotein secretion is not altered in patients with peroxisome biogenesis disorders [42]. Although expression of genes encoding for enzymes controlling β-oxidation was increased in *Pex16* KO mice, we cannot exclude reduced β-oxidation in these mice. As mitochondrial function was decreased in the *Pex16* KO mice fed an LPD, this is likely the most critical contributor to the exacerbated steatosis in these mice.

### Decreased autophagy and induction of mitochondria biogenesis contribute to increased mitochondrial mass in *Pex16* KO mice

Our studies suggest that the increase in mitochondrial mass might be caused by two distinct mechanisms. First, the activation of AMPK by functional loss of peroxisome (Fig. 3) could induce mitochondrial biogenesis via the AMPK-dependent activation of PGC-1α [43]. The increase in activated pAMPK also inhibits mTORC1 and activates autophagy [44]. We found pAMPK to be increased in livers of *Pex16* KO, a result similar to that described in liver-specific PEX5 deficiency [45]. Second, our autophagy assays suggest that hepatocyte autophagic flux is decreased in *Pex16* KO mice. We speculate that this increased mitochondrial biogenesis and reduced autophagic flux are compensatory mechanisms that preserve mitochondrial function by increasing mitochondrial mass. Down-regulating β-oxidation could also prevent further mitochondrial damage by limiting the production of ROS.

### Loss of functional peroxisomes exacerbates low protein diet induced mitochondrial changes through separate pathways

Nutritional stress induced by LPD in weanlings exacerbated the hepatic steatosis caused by *Pex16* deficiency. Interestingly, cold-induced stress also exacerbates mitochondrial damage in brown adipocytes in adipose-specific *Pex16* KO mice [46]. The small mitochondria seen in the livers of the *Pex16* KO mice were not described in those of the *Pex5* deficient model [12], and this might be related to potential differential effects on mitochondrial homeostasis where PEX16 is involved in the early steps of peroxisome biogenesis, whereas PEX5 imports peroxisomal proteins. Interestingly, LPD feeding led to a fewer and smaller mitochondria compared to normal chow diet animals, associated with signs of reduced biogenesis and despite possibly lower autophagy activation. These findings are contrary to those described in a rat model of LPD-induced loss of functional peroxisomes and hepatic mitochondrial dysfunction where an accumulation of damaged mitochondria was observed with LPD feeding [17]. This discrepancy might relate to differences in severity of the two model systems, or a temporal difference as the rats were placed on an LPD for 4 weeks instead of the 2 weeks in the mice.

### Fibrates improve mitochondrial function in *Pex16* KO low protein fed mice

PPARα induces peroxisome and mitochondrial biogenesis, increases β-oxidation, and has recently been shown to induce autophagy [36]. Previously, we demonstrated that activation of PPARα could reverse peroxisome loss and improve mitochondria fitness in the hepatocytes of rats subjected to LPD [17]. Here, we examined whether peroxisomes are necessary for the possible rescue of mitochondrial function by PPARα induction. Fibrate treatment up-regulated genes involved in peroxisome biogenesis (Fig. 7a) and peroxisomal and mitochondrial β-oxidation, and this was associated with reduced lipids in both LPD-fed *Pex16* KOs and wild-type mice, suggesting a peroxisome-independent effect. Fibrates also increased mitochondrial biogenesis, which is consistent with recent observations [47]. Fibrate treatment also led to an induction of autophagy as indicated by decreased mTORC1 activity, increased LC3B-II, and decreased p62. Activation of PPARα has been shown to up-regulate autophagy transcriptionally [36], and this could account, in part, for the increased autophagy in our model. Of interest, fibrate treatment has been piloted in patients with Zellweger Syndrome but without clear clinical impact [48, 49]. The lack of effect in patients could be related to the possibility that the disease state was already too advanced for these patients with Zellweger syndrome to benefit from fibrate treatment, or it is also possible that mitochondrial damage may not be the sole pathology of Zellweger syndrome.

**Fig. 9:**
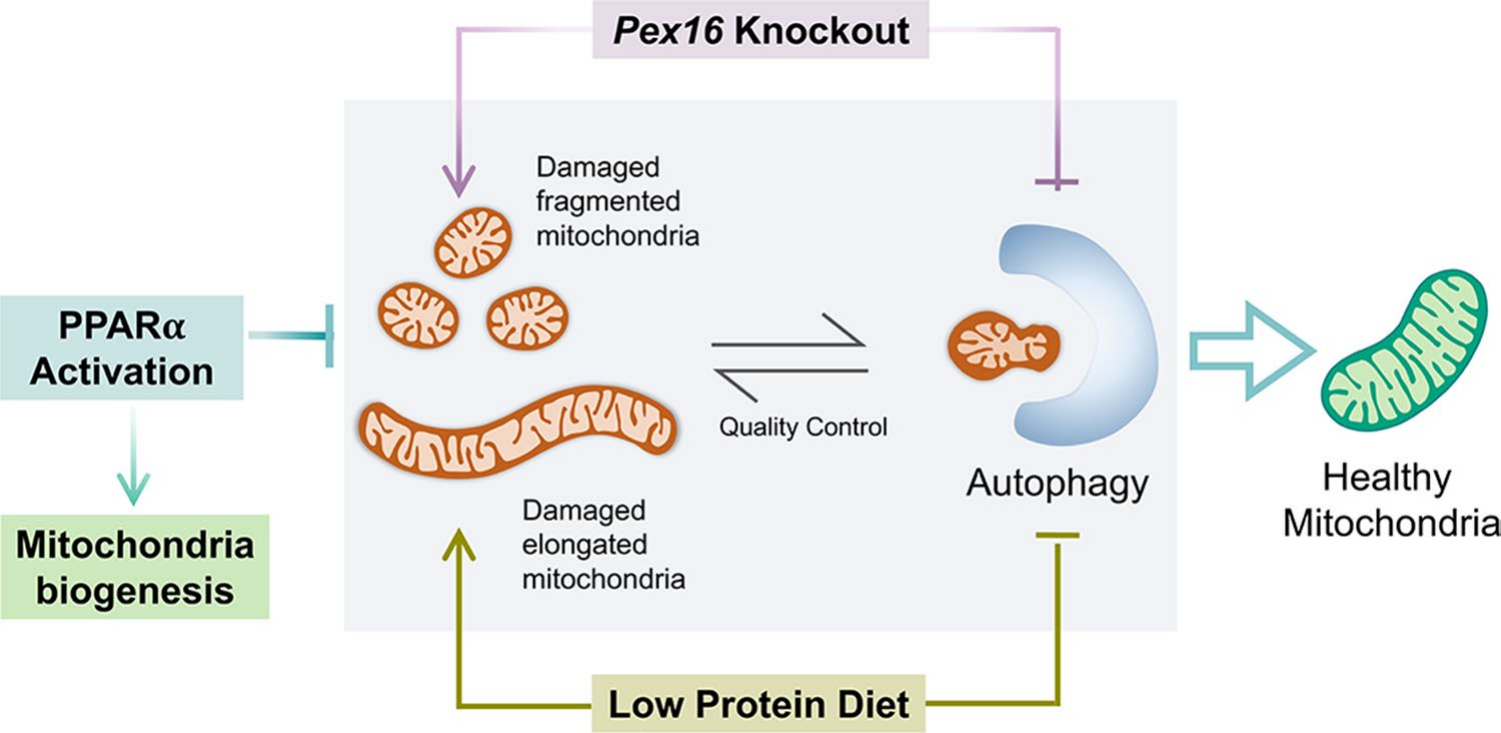
Proposed mechanistic model by which *Pex16* loss and LPD lead to damaged mitochondria and reduced autophagy. Loss of hepatic *Pex16* leads to fragmented mitochondria. Feeding a low protein diet leads to elongated mitochondria. A low protein diet in mice with peroxisomal defects impacts mitochondrial biogenesis and autophagy. Activation of the PPARα pathway restores mitochondrial biogenesis and hepatic mitochondrial function in low protein-fed mice, even in the absence of functional peroxisomes

In conclusion, this study describes the phenotypical consequences of hepatic loss of *Pex16*, which are exacerbated by nutritional stress induced by LPD. We demonstrated that *Pex16* deficiency leads to enhanced mitochondrial biogenesis and reduced autophagy resulting in an increase in mitochondrial mass (Fig. 9), in order to compensate for the loss of the peroxisomal protective role. These mitochondria do maintain their respiration potential but are more susceptible to metabolic stress-induced damage. PPARα treatment improved mitochondrial health by overcoming the reduction in autophagy flux caused by the loss of PEX16 and by enhancing mitochondrial biogenesis. This improvement is independent of the peroxisome function. Our finding that mitochondrial dysfunction can be uncoupled from peroxisome loss is of relevance for children with peroxisomal biogenesis disorders and suggest that interventions to restore mitochondrial homeostasis and preventing additional stressors such as malnutrition could improve their outcome.

### Supplementary Information

Below is the link to the electronic supplementary material.

Supplementary file1 (DOCX 6547 KB)

## Data Availability

The datasets generated during and/or analyzed during this study are available in the BioStudies repository, https://www.ebi.ac.uk/biostudies, accession number S-BSST100.

## Abbreviations

Acaa1a: Acetyl-coenzyme A carboxylase carboxyl transferase subunit α
Acaca: Acetyl-CoA carboxylase α
Acadm1: Acyl-CoA dehydrogenase medium chain 1
Acadv1: Very long-chain specific acyl-CoA dehydrogenase 1
Acadl: Acyl-CoA dehydrogenase long chain
Acca2: Acetyl-CoA carboxylase α subunit
Acot12: Acyl-CoA thioesterase 12
Acox1: Peroxisomal acyl-coenzyme A oxidase 1
Acsl1: Acyl-CoA synthetase long chain family member 1
ADP: Adenosine diphosphate
AMP: Adenosine monophosphate
AMPK: AMP-activated protein kinase
ATP: Adenosine triphosphate
BSA: Bovine serum albumin
CCM: Central carbon metabolism
CQ: Chloroquine
COX IV: Cytochrome *c* oxidase subunit 4
DAPI: 4′,6-Diamidino-2-phenylindole
DRP1: Dynamin-1-like protein
DMSO: Dimethyl sulfoxide
Ech1: Enoyl-CoA hydratase 1fabp1
ER: Endoplasmic reticulum
ESCs: Mouse embryonic stem cells
ETC: Electron transport chain
4EBP1: Eukaryotic translation initiation factor 4E binding protein 1
Fabp1: Fatty acid binding protein 1
Fasn: Fatty acid synthase
FCCP: Carbonyl cyanide-4 (trifluoromethoxy) phenylhydrazone
FF: Fenofibrate
GMP: Guanosine monophosphate
GDP: Guanosine diphosphate
GTP: Guanosine triphosphate
H&E: Haemotoxylin and eosin
HSP60: Heat shock protein 60
IP: Intraperitoneal
KO: Knockout
LC3B: Microtubule-associated protein light chain 3B
LPD: Low protein diet
MFN2: Mitofusin-2
mtDNA: Mitochondrial DNA
NAD: Nicotinamide adenine dinucleotide
NADH: Nicotinamide adenine dinucleotide hydride
NADPH: Nicotinamide adenine dinucleotide phosphate
NRF1: Nuclear respiratory factor 1
OCR: Oxygen consumption rat
PBS: Phosphate-buffered saline
PCA: Perchloric acid
PEP: Prolyl endopeptidase
PINK1: PTEN induced kinase 1
PMP70: Peroxisomal membrane protein 70
p62/SQSTM1: Autophagy adaptor protein sequestosome 1
p70S6K: Ribosomal protein S6 kinase β-1
PEX: Peroxin
PPARGC1A: Peroxisome-proliferator-activated receptor coactivator-1α
PPARα: Peroxisome proliferator activated receptor α
Rpl13a: Ribosomal protein L13a
TBS: Tris-buffered saline
TCA: Tricarboxylic acid
TEM: Transmission electron microscopy
TOM20: Membrane translocase 20
UMP: Uridine monophosphate
UDP: Uridine diphosphate
UTP: Uridine triphosphate
