# Supplementary material for: Loss of functional peroxisomes leads to increased mitochondrial biogenesis and reduced autophagy that preserve mitochondrial function
Source: Cell Mol Life Sci. 2023 Jun 20;80(7):183. doi: 10.1007/s00018-023-04827-3 (PMC10281899; doi:10.1007/s00018-023-04827-3)
Supplement: Supplementary file 1 — Supplementary file1 (DOCX 6547 KB) [file 18_2023_4827_MOESM1_ESM.docx]

**Supplementary Information**

**Loss of functional peroxisomes leads to increased mitochondrial biogenesis and reduced autophagy that preserve mitochondrial function**

Lijun Chi^1,†^, Dorothy Lee^1,2,†^, Sharon Leung^3^, Guanlan Hu^1,4^, Bijun Wen^1,4^, Paul Delgado-Olguin^1,5,6^, Ren Li^3^, John H Brumell^3,5^, Peter K Kim^3,7*^, Robert H J Bandsma^1,4,8,9*^

^1^ Translational Medicine program, The Hospital of Sick Children, Toronto, ON, M5G 0A4, Canada

^2^ Department of Physiology, University of Toronto, Toronto, ON, M5S 1A1,Canada

^3^ Cell Biology program, The Hospital of Sick Children, Toronto, ON, M5G 0A4,Canada

^4^ Department of Nutritional Sciences, University of Toronto, Toronto, ON, M5S 1A1, Canada

^5^ Department of Molecular Genetics, University of Toronto, Toronto, ON, M5S 1A1, Canada

^6^ Heart & Stroke Richard Lewar Centre of Excellence, Toronto, Ontario, M5S 3H2, Canada.

^7^ Department of Biochemistry, University of Toronto, Toronto, ON, M5S 1A1, Canada

^8^ Centre for Global Child Health, The Hospital of Sick Children, Toronto, ON, M5G 0A4, Canada

^9^ Division of Gastroenterology, Hepatology and Nutrition, The Hospital for Sick Children, Toronto, ON, M5G 0A4, Canada

^†^ These authors contributed equally as first authors.

***Corresponding authors:**

Peter Kim, Cell Biology Program, The Hospital for Sick Children, Peter Gilgan Centre for Research and Learning, 686 Bay Street, Toronto, ON M5G 0A4, Canada. Tel.: +1 416 813 5983; +1 416 813 5028. Email address: pkim@sickkids.ca

Robert Bandsma, Translational Medicine Program, The Hospital for Sick Children, Peter Gilgan Centre for Research and Learning, 686 Bay Street, Toronto, ON M5G 0A4, Canada. Tel.: +1 4168137654x9057; fax: +1 4168134972 (R.H.J. Bandsma).

E-mail addresses: robert.bandsma@sickkids.ca (R.H.J. Bandsma)


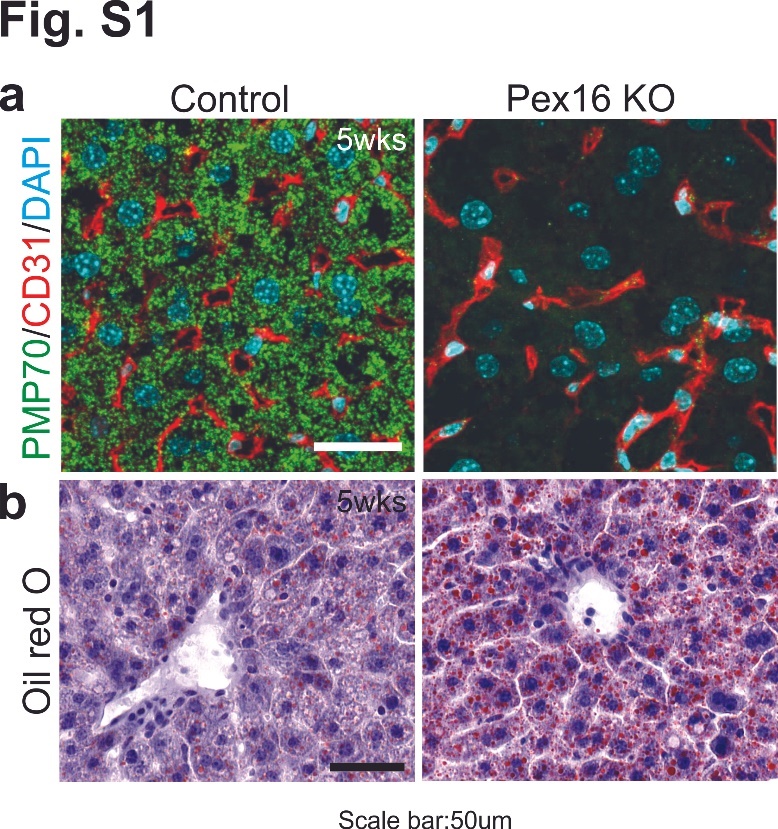


**Fig. S1. Complete loss of peroxisomes by 5 weeks in *Pex16* KO mice.**

**a** Immunofluorescence of PMP70 and CD31 stained in wild-type and *Pex16* KO liver at 5 weeks. **b** Oil red O staining in control and *Pex16* KO liver. n=6 mice per group. Scale bar: 50 µm.


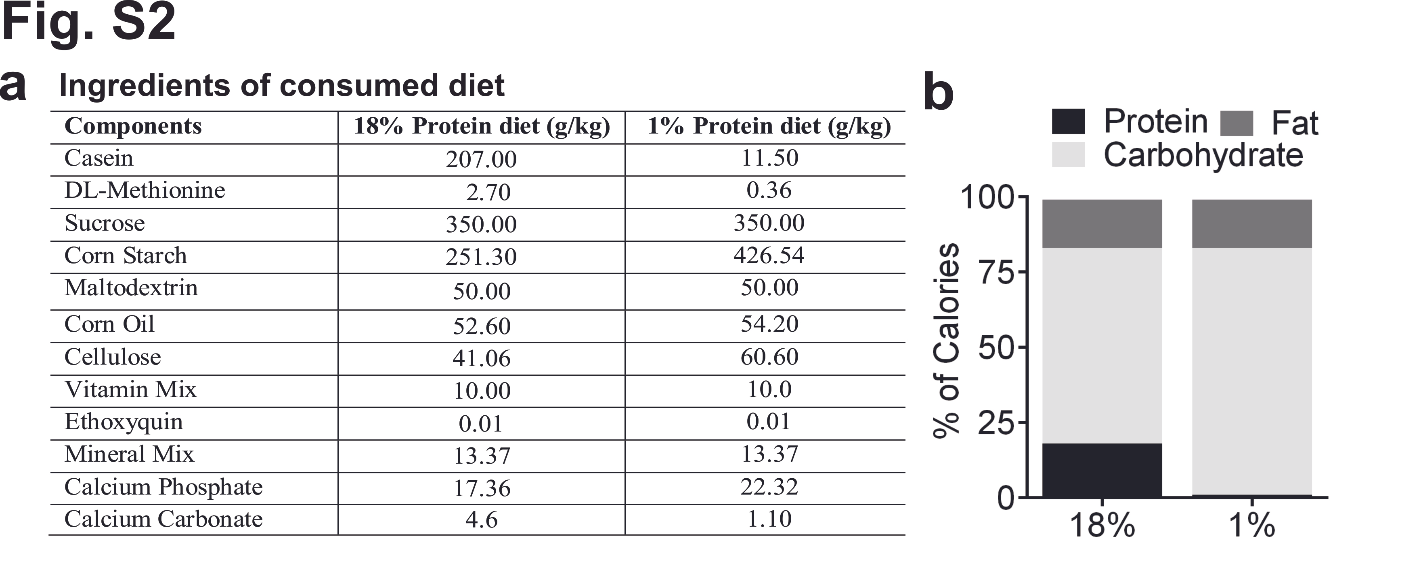
**Fig. S2 Ingredients of the consumed diets.**

**a** The details of the diet components. **b** Graph shows the percentage of protein, fat and carbohydrate.


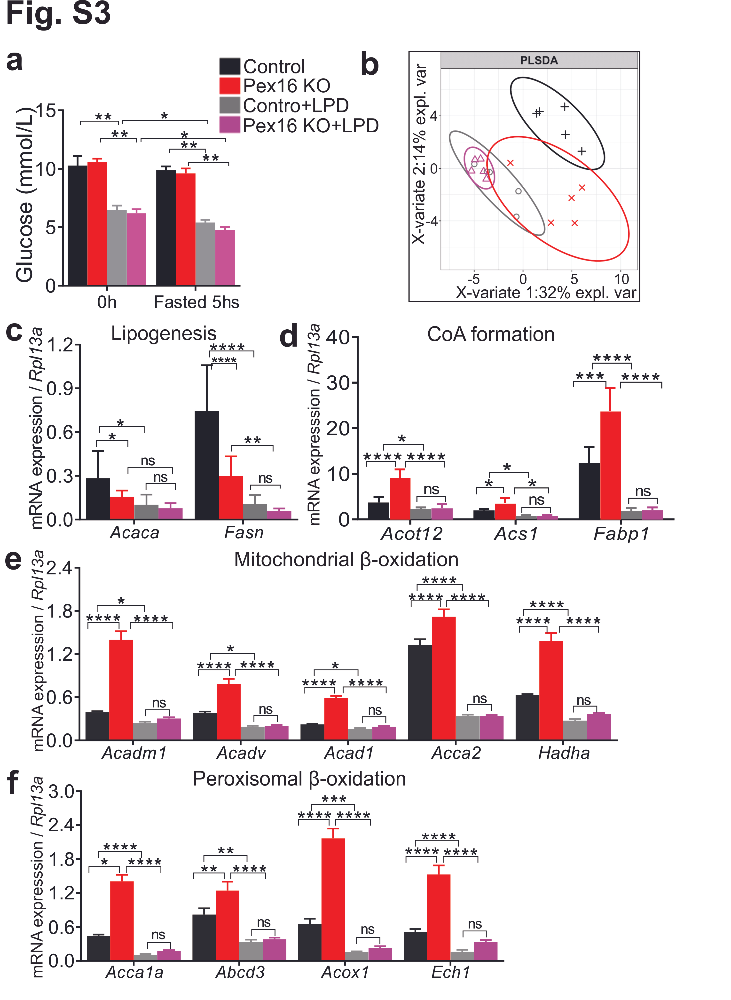


**Fig. S3 Central carbon metabolism (CCM) hepatic metabolite plots and expression of genes involved in lipogenesis and** $\beta$**-oxidation.**

**a** Blood glucose test in mice non-fasted and fasted for 5 hours. **b** PLS-DA plot displaying the separation of CCM assay results. **c** qPCR analysis of lipogenesis markers. **d** qPCR shows the expression of genes involved in CoA formation. **e** qPCR shows the expression of genes involved in mitochondrial $\beta$-oxidation. **f** qPCR shows the expression of genes involved in peroxisomal $\beta$-oxidation. Data are represented as mean ± SEM, n=6-8 mice per group. *p<0.05, **p<0.01, ***p<0.001, ****p<0.0001.


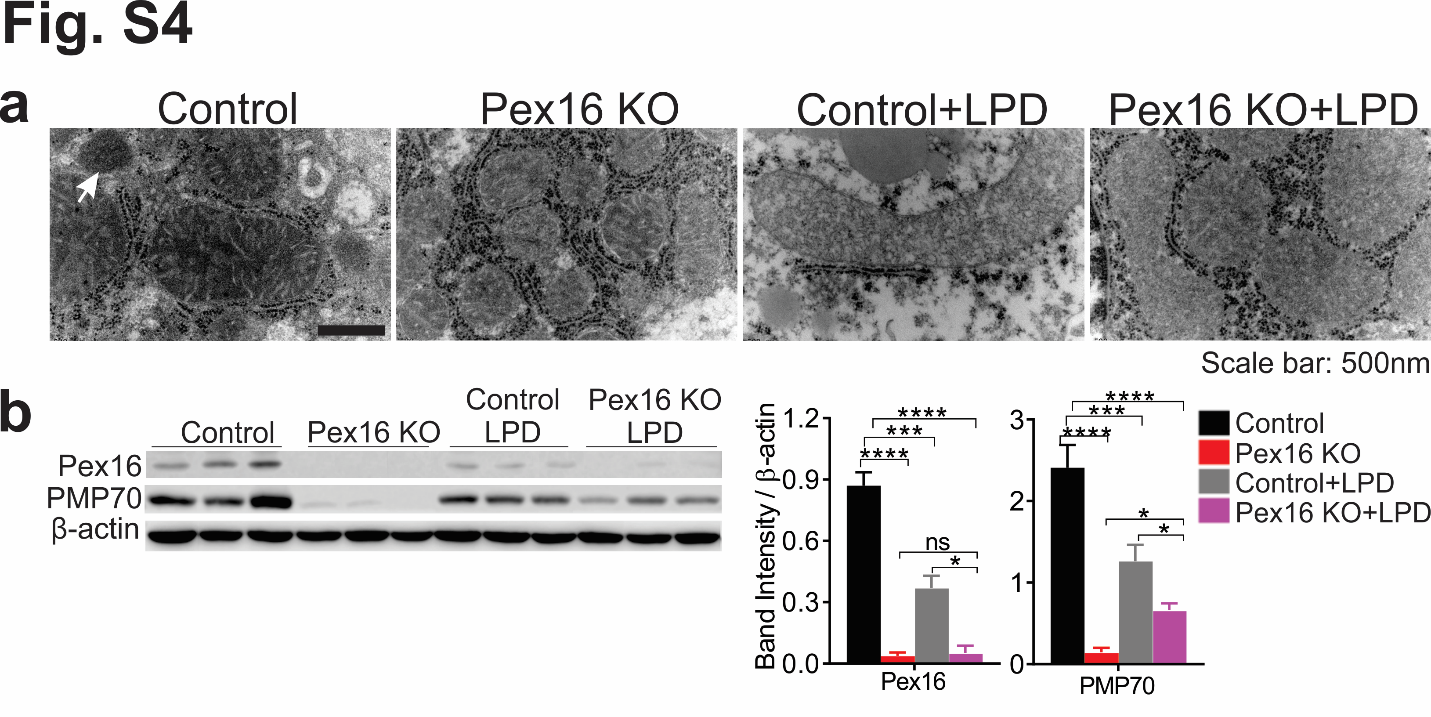


**Fig. S4 Mitochondrial ultra-structure of TEM images in livers from LPD-fed mice.**

**a** Mitochondrial ultra-structure shown as TEM images (50,000x).  **b** Western blot of PEX16 and PMP70, and quantification of band intensity relative to $\beta$-actin. Data are represented as mean ± SEM, n=6-8 mice per group. Scale bar: **a** 500 nm. *p<0.05, ***p<0.001, ****p<0.0001.


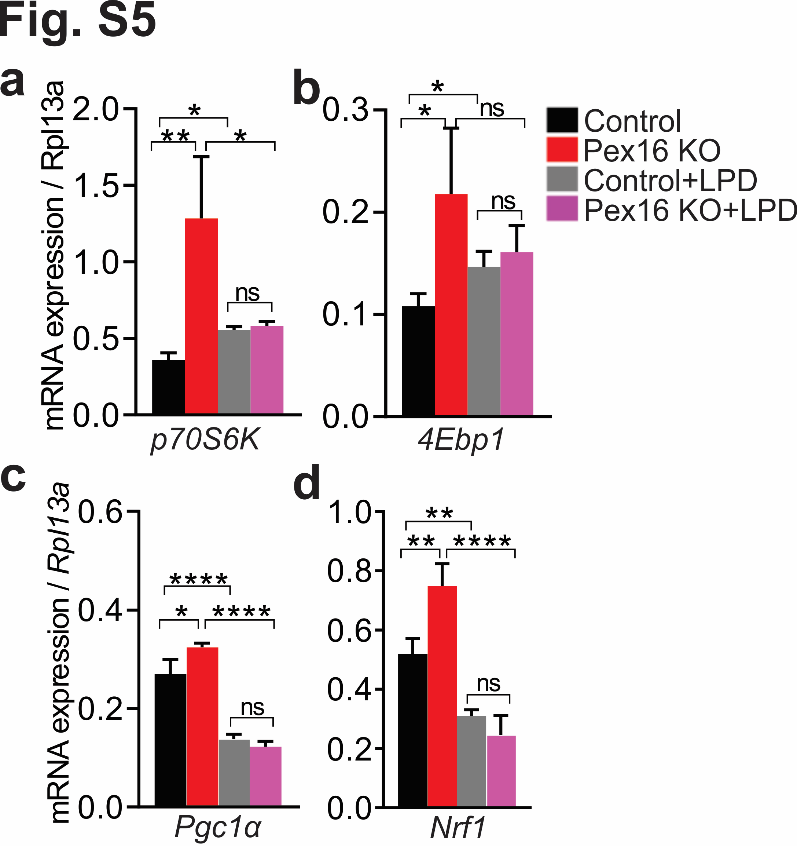


**Fig. S5 Loss of *Pex16* and LPD feeding affect mRNA level of autophagy markers and biogenesis markers.**

**a** qPCR analysis of mitochondrial autophagy marker *p70S6K*. **b** qPCR analysis of mitochondrial autophagy marker *4Ebp1*. **c** qPCR analysis of mitochondrial biogenesis marker *Pgc1α*.  **d** qPCR analysis of mitochondrial biogenesis marker *Nrf1*. Data are represented as mean ± SEM, n=6 mice per group. *p<0.05, **p<0.01, ****p<0.0001


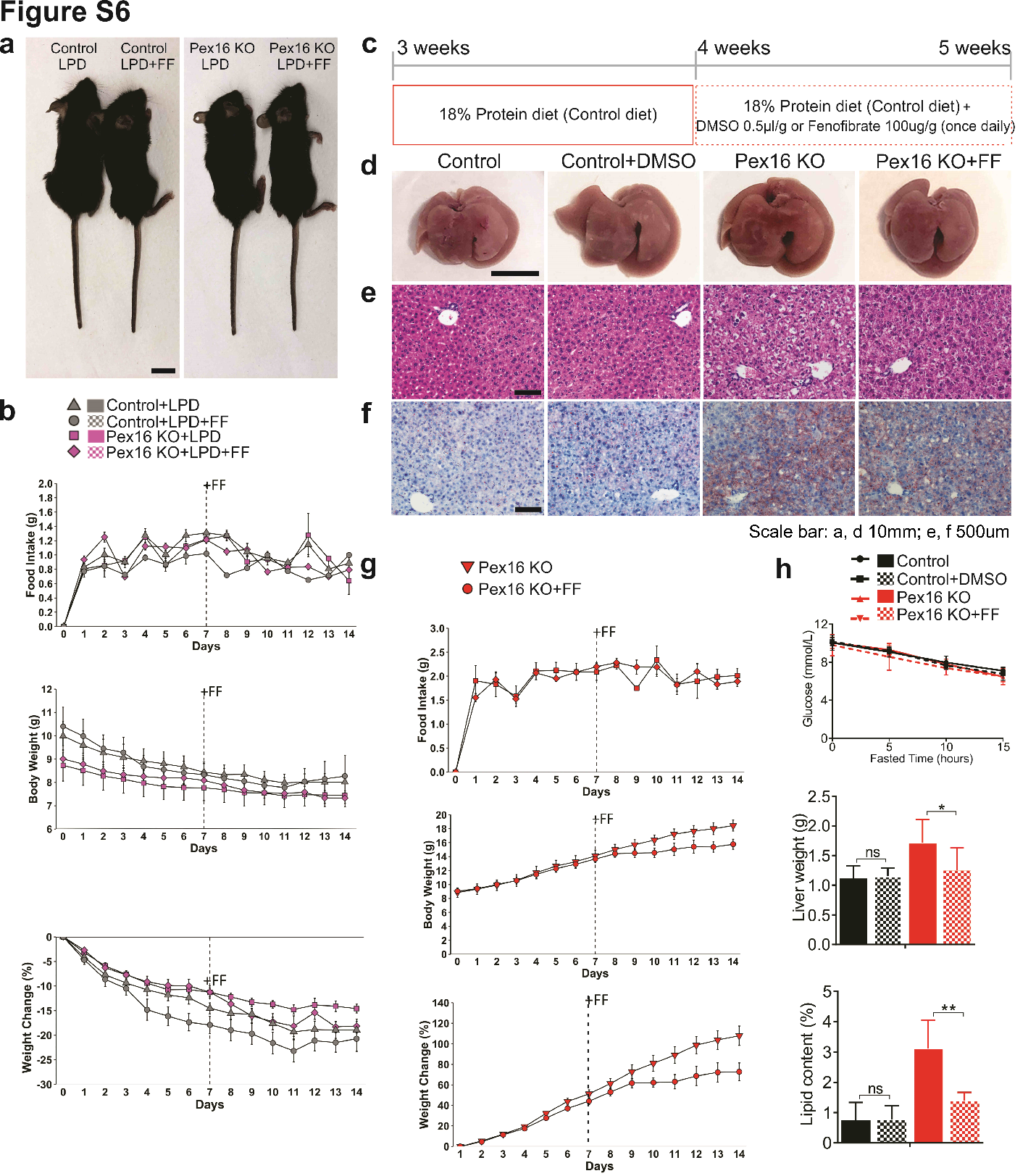


**Fig. S6 Body characteristics, food intake and histology in different mouse groups.**

**a** Body morphology in LPD and fenofibrate treated mice. **b** Food intake, body weight and percentage body weight change in LPD diet groups. **c** Schematic of 18% protein control diet-fed mice gavage with DMSO and fenofibrate. **d** Liver morphology of wild type and *Pex16* KO mice on a 18% protein diet. **e** H&E staining of wild type and *Pex16* KO mice on a 18% protein diet. **f** Oil red O staining in sections of wild type and *Pex16* KO mice on a 18% protein diet. **g** Food intake, body weight and percentage body weight change of wild type and *Pex16* KO mice on a 18% protein diet. **h** Blood glucose test, liver weight and lipid droplets content. Data are represented as mean ± SEM, n=6-8 mice per group. Scale bar: **a, d** 10mm, **e, f** 500 µm. *p<0.05, **p<0.01. FF, fenofibrate.


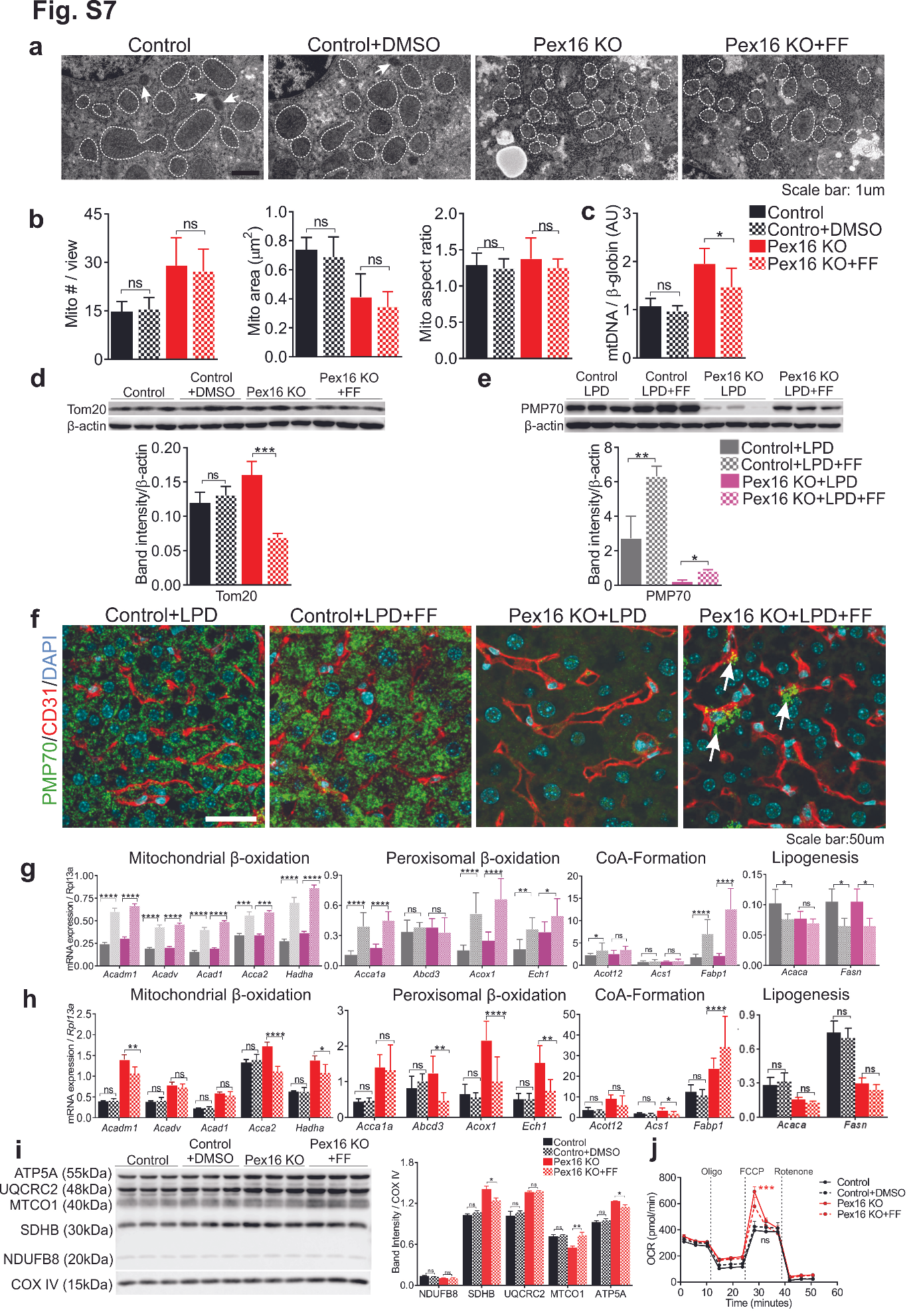


**Fig. S7 Mitochondrial ultra-structure and measurement of mitochondrial respiration in control diet-fed mice and mRNA levels of β-oxidation markers in LPD diet groups treated with fenofibrate.**

**a** TEM images (20,000x) of different control diet fed mouse groups. **b** Quantification of mitochondrial number, mitochondrial area, and mitochondrial aspect ratio (length/width) from TEM images. **c** qPCR analysis on mtDNA. **d** Western blot of hepatic mitochondrial Tom20, and quantification of band intensity relative to $\beta$-actin. **e** Western blot of hepatic PMP70 in LPD diet groups treated with fenofibrate, and quantification of band intensity relative to $\beta$-actin. **f** Immunofluorescence of PMP70 and CD31 stained in livers of control diet fed groups, arrows indicate PMP70+ CD31+ double positive staining. **g** qPCR analysis on expression of enzymes of mitochondrial $\beta$-oxidation and lipogenesis markers in LPD diet groups treated with fenofibrate. **h** qPCR analysis of enzymes of mitochondrial $\beta$-oxidation and lipogenesis markers in control diet-fed mice. **i** Western blots of mitochondrial electron transport chain (ETC) complexes subunits, NDUFB8 (Complex I), SDHB (Complex II), UQCRC2 (Complex III), MTCO1 (Complex IV) and ATP5A (Complex V) from isolated hepatic mitochondria, quantification of band intensity relative to COX IV. **j** Hepatocyte mitochondrial respiration determined by oxygen consumption rate (OCR). Black asterisks indicate significance between control vs control+DMSO liver, red asterisks indicate significance between *Pex16*KO vs *Pex16*KO+FF liver. Data are represented as mean ± SEM, n=6 mice per group. Scale bar: **a** 1 um, **f** 50um. *p<0.05, **p<0.01, ***p<0.001, ****p<0.0001. FF, fenofibrate.


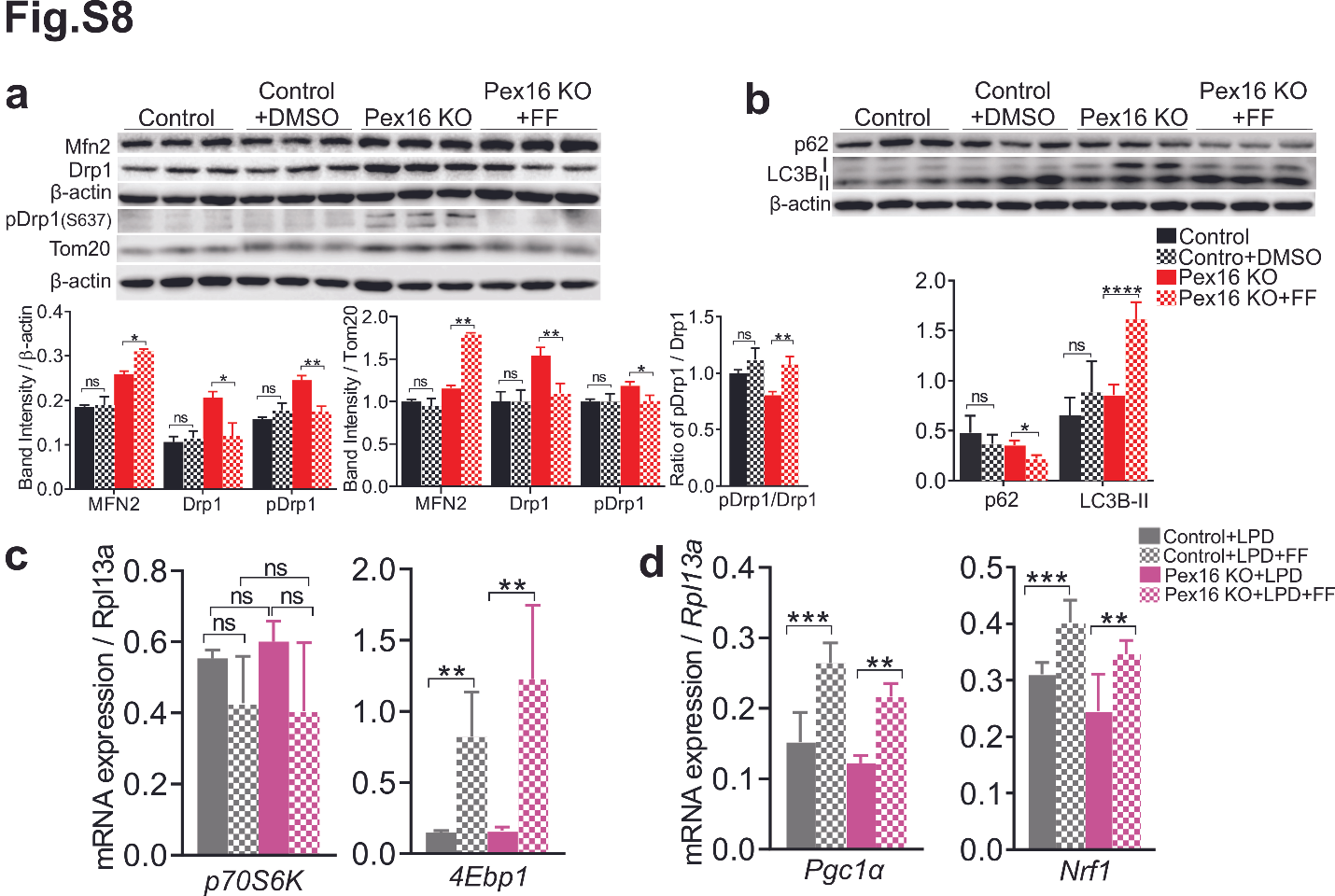


**Fig. S8 Mitochondrial homeostasis in control diet-fed mice and mRNA levels of mTOR effectors in LPD diet groups treated with fenofibrate.**

**a** Western blot of hepatic mitochondrial Mfn2, Drp1, and pDrp1 in control diet fed groups, and quantification of band intensity relative to $\beta$-actin and Tom20, and the ratio of pDrp1 to total Drp1. **b** Western blot of autophagy markers p62, LC3B, and quantification of band intensity relative to $\beta$-actin. **c** qPCR analysis on expression of mTOR effectors *p70S6K*, *4Ebp1* in LPD diet groups. **d** qPCR analysis on expression of biogenesis markers *Pgc1α* and *Nrf1* in LPD diet groups. Data are represented as mean ± SEM, n=6 mice per group. *p<0.05, **p<0.01, ***p<0.001, ****p<0.0001. FF, fenofibrate.

**Table S1. Primers used to genotype the mouse lines**

**Mouse lines Primers 5’-3’ Fragment (bp)**

*Pex16* 5’-GGGCACTGTTGTTCTGTCTAGCTGAAGG-3’ WT: 591

5’-TGGCAAAGTTCTCACCACAGGCAAC-3’ Flox: 720

*Albumin-Cre* 5’-ACCGTCAGTACGTGAGATATCTT-3’

5’-ACCGTCAGTACGTGAGATATCTT-3’ 320

**S2. Antibodies used for Immunofluorescence and western blotting**

**Antibody Source Identifier**

4EBP1 (1:1000) Cell Signaling Technology 9644

p4EBP1 (1:500) Cell Signaling Technology 2855 AMPKα (1:1000) Cell Signaling Technology 2532

p-AMPK α (1:1000) Cell Signaling Technology 4184

β-actin (1:2000) Abcam Biotechnology ab8227

BODIPY (1:1000) Invitrogen D392

CD31 (1:1000) BD Biosciences 553370

COX IV (1:1000) Abcam Biotechnology ab14748

DRP1 (1:1000) Abcam Biotechnology ab56788

pDRP1(S637) (1:2000) Abcam Biotechnology ab193216

GAPDH (1:2000) Novus Biotechnology NB-300-221

HSP60 (1:1000) Abcam Biotechnology ab46798

LC3B (1:1000) Cell Signaling Technology 2775

MFN2 (1:1000) Abcam Biotechnology ab56889

NRF1(1:500) Abcam Biotechnology ab34682

p62/SQSTM1(1:1000) Novus Biotechnology NBP1-48320

p70S6K (1:1000) Cell Signaling Technology 2708

p-p70S6K (1:1000) Cell Signaling Technology 9234

Pex16 (1:1000) Abcam Biotechnology ab230303

PGC-1α (1:1000) Abcam Biotechnology ab54481

PINK1(1:500) NOVUS Biotechnology NB100-493

PMP70 (1:1000) Abcam Biotechnology ab3421

PPARα (1:1000) Santa Cruz Biotechnology sc-9000

TOM20 (1:1000) Santa Cruz Biotechnology sc-11415

Total OXPHOS Cocktail (1:1000) Abcam Biotechnology ab110413

DAPI (1:1000) Sigma D9564

Donkey anti-mouse IgG-HRP(1:2000) Santa Cruz Biotechnology sc-2314

Mouse anti-rabbit IgG-HRP (1:2000) Santa Cruz Biotechnology sc-2357

Goat anti-rabbit Alexa Fluor 568(1:1000) Thermo Fisher Scientific A11011

Goat anti-mouse Alexa Fluor 488(1:1000) Thermo Fisher Scientific A28175

**Table S3. Primers used for qPCR**

**Gene Forword primers Reverse primers**

mtDNA CCCAGCTACTACCATCATTCAAGT GATGGTTTGGGAGATTGGTTGATGT

b-globin AAGGTGAACGCCGATGAAGT ATCAAAGTACCGCTGGGTCC

Acadm1 TGACAAAAGCGGGGAGTACC CCATACGCCAACTCTTCGGT

Acadvl GTAGCCTCCATCCGAAGCTC CAGGCCCCCATTACTGATCC

Acadl TGCACACATACAGACGGTGC CATGGAAGCAGAACCGGAGT

Acca2 CCTGCTACGAGGTGTGTTCA GAAGTCCTTGAGAAGGCCCC

Abcd3 TCCCCGTGCATGTCAACTTT TTTCTTACTTAGCGTTCAGTGGC

Acox1 CATGTGGTTTAAAAACTCTGTGC GGCATGAAGAAACGCTCCTG

Acaa1a ACATCTCCGTGGGCAATGTT CTCAGAAATTGGGCGATGCG

Acot12 AGCAATTCGTGCACCGTAAC AAGTCCATCAGGAGTAGCATTC

Acsl1 GCCGCGACTCCTTAAATAGCA ATGCAGAATTCTCCTCCGCTG

Acaca CGATCTATCCGTCGGTGGTC GGTCTGCCATCTTAATGTATTCTGC

Ech1 CAGCTAAACCGGCCAGAGAA GGCAGTCCGAGTCTTTGGAT

Fabp1 AGTCAAGGCAGTCGTCAAGC ATGTCGCCCAATGTCATGGT

Fasn TGCACCTCACAGGCATCAAT GTCCCACTTGATGTGAGGGG

PGC-1a AAAAAGCTTGACTGGCGTCAT AGAGGCTGGTCCTCACCAA

Hadha AGTGGAAAGCGTGACTCCAG GTAGTGCATGCCGATCACCT

Nrf1 AAAAAGCTTGACTGGCGTCAT AGAGGCTGGTCCTCACCAA

p70S6K GGCTCGGAAGGTGGAACCTC TCAGGGCTGTCAACAGGTGTC

4Ebp1 CTAGCCCTACCAGCGATGAG CCTACGGCTGGTCCCTTAAA

Rpl13a TCCCTCCACCCTATGACAAG GTCACTGCCTGGTACTTCC

**Table S4. Central carbon metabolism (CCM) analysis**

| **Mean±SEM** | **Control*** | **Pex16 KO*** | **Control+LPD*** | **Pex16 KO+LPD*** |
| --- | --- | --- | --- | --- |
| **AMP** | 6.793±1.851 | 24.109±7.315 | 3.892±1.817 | 1.845±0.093 |
| **ADP** | 4.016±1.028 | 4.952±0.964 | 3.691±1.101 | 1.194±0.192 |
| **ATP** | 1.771±0.204 | 1.583±0.287 | 1.833±0.207 | 1.886±0.394 |
| **GMP** | 14.515±8.413 | 48.186±13.157 | 0.649±0.185 | 0.419±0.046 |
| **GDP** | 0.492±0.080 | 0.456±0.064 | 0.422±0.091 | 0.237±0.042 |
| **GTP** | 0.110±0.033 | 0.081±0.013 | 0.124±0.014 | 0.103±0.022 |
| **UMP** | 60.783±32.349 | 57.197±17.906 | 30.750±24.008 | 1.240±0.198 |
| **UDP** | 0.358±0.144 | 0.678±0.192 | 0.289±0.170 | 0.030±0.007 |
| **UTP** | 2.799±0.627 | 2.519±0.311 | 2.428±0.890 | 2.177±0.407 |
| **cyclic-ADP-ribose** | 1.489±0.319 | 1.232±0.137 | 0.930±0.084 | 0.669±0.035 |
| **cyclic-AMP** | 0.027±0.009 | 0.017±0.002 | 0.014±0.002 | 0.012±0.001 |
| **6-P-Gluconate** | 10.118±1.073 | 7.342±1.370 | 4.679±0.737 | 4.622±0.608 |
| **Acetylglucosamine-1P** | 3.916±0.517 | 2.659±0.152 | 3.809±0.915 | 3.889±0.358 |
| **Acetyl-Phosphate** | 51.466±20.141 | 142.745±43.656 | 54.391±23.632 | 30.505±4.236 |
| **ADP-Glucose** | 0.229±0.082 | 0.155±0.036 | 0.127±0.078 | 0.007±0.002 |
| **2,3-bisP-Glycerate** | 0.160±0.038 | 0.066±0.008 | 0.061±0.026 | 0.112±0.012 |
| **DHAP** | 8.262±4.384 | 18.997±5.063 | 8.580±4.848 | 3.361±0.515 |
| **Total of Fructose-bisP/Glucose-1,6-bisP** | 1.932±1.537 | 2.319±1.157 | 1.217±0.814 | 0.297±0.095 |
| **Fructose-6P** | 78.884±32.734 | 199.753±64.913 | 97.355±42.011 | 45.277±7.299 |
| **Glucosamine-6P** | 4.751±1.626 | 4.295±2.290 | 2.860±1.007 | 1.511±0.259 |
| **Glycerate-2- or 3-P** | 9.324±4.561 | 8.000±6.807 | 14.458±5.067 | 14.983±3.808 |
| **Glycerol-3-P** | 13.555±5.793 | 7.994±1.589 | 6.891±1.311 | 20.955±9.679 |
| **PEP** | 5.133±2.278 | 4.064±3.328 | 6.237±1.875 | 5.721±1.272 |
| **Phosphocreatine** | 0.036±0.022 | 0.027±NA | 0.098±0.023 | 0.025±0.007 |
| **Ribulose-1,5-bisP** | 0.340±0.099 | 0.324±0.076 | 0.228±0.056 | 0.169±0.027 |
| **Ribulose-5P** | 495.318±296.239 | 1142.994±288.208 | 470.129±284.020 | 62.926±8.595 |
| **Sedoheptulose-7P** | 43.729±24.732 | 14.936±4.174 | 25.966±11.792 | 103.365±19.443 |
| **UDP-Glucose** | 64.367±25.932 | 86.923±32.916 | 33.122±27.000 | 1.775±0.499 |
| **Acetyl-CoA** | 0.006±0.001 | 0.006±0.002 | 0.002±0.000 | 0.001±0.000 |
| **Hs-CoA** | 0.399±0.064 | 0.261±0.047 | 0.152±0.049 | 0.120±0.030 |
| **Malonyl-CoA** | 0.003±0.000 | 0.005±0.001 | 0.003±0.001 | 0.002±0.000 |
| **Succinyl-CoA** | 1.347±0.364 | 0.181±0.031 | 0.138±0.017 | 0.058±0.013 |
| **NAD+** | 0.764±0.221 | 0.398±0.109 | 0.413±0.163 | 0.120±0.031 |
| **NADH** | 0.540±0.131 | 0.850±0.049 | 0.586±0.065 | 0.523±0.168 |
| **NADP+** | 0.225±0.030 | 0.331±0.067 | 0.200±0.047 | 0.162±0.034 |
| **NADPH** | 0.019±0.002 | 0.016±0.002 | 0.006±0.001 | 0.005±0.001 |
| **Erythrose-4P** | 0.888±0.136 | 1.147±0.193 | 0.547±0.030 | 0.670±0.100 |
| **Glucose** | 6941.000±916.244 | 4818.000±486.882 | 2719.200±388.446 | 2717.000±460.545 |
| **Glucose-6P** | 343.300±110.285 | 241.452±103.454 | 65.242±8.420 | 59.036±8.155 |
| **Glycerylaldehyde-3P** | 75.176±5.477 | 87.111±31.639 | 6.148±0.922 | 8.848±1.238 |
| **Mannose-6P** | 72.756±21.025 | 60.618±26.580 | 14.162±4.024 | 18.124±2.358 |
| **Ribose-5P** | 90.956±10.147 | 140.030±26.699 | 23.272±1.858 | 20.878±3.415 |
| **α-Hydroxyglutaric acid** | 30.630±9.137 | 15.041±2.630 | 6.124±0.704 | 7.314±1.705 |
| **α-Ketoglutaric acid** | 11.510±2.050 | 7.288±1.843 | 3.515±0.692 | 2.628±1.020 |
| **Citric acid** | 23.182±3.150 | 8.398±3.064 | 41.552±36.686 | 54.330±29.267 |
| **Fumaric acid** | 791.120±74.906 | 799.260±101.674 | 1061.000±126.879 | 894.520±197.046 |
| **Glycolic acid** | 45.936±6.260 | 38.522±3.542 | 39.074±6.105 | 80.960±25.563 |
| **Isocitric acid** | 1.520±0.375 | 1.430±0.252 | 2.304±1.373 | 4.434±1.353 |
| **Lactic acid** | 9314.600±1249.930 | 10124.400±1173.850 | 5352.600±746.665 | 5183.800±1317.818 |
| **Malic acid** | 1204.820±185.059 | 1231.020±208.121 | 1719.200±149.080 | 1475.800±266.543 |
| **Pyruvic acid** | 61.132±13.493 | 62.642±14.255 | 53.864±5.517 | 58.894±9.722 |
| **Succinic acid** | 7.218±1.229 | 19.328±8.808 | 6.140±1.201 | 6.124±1.324 |

*****All values are expressed as the mean±SEM, versus the WT control group

**Table S5. Statistical analyses of the CCM data**

| **Diet group** | **Name** | **p-value** | **p-value (FDR)** | **SiSignificance (FDR)*** |
| --- | --- | --- | --- | --- |
| Pex16 KO+LPD | AMP | 0.282 | 0.551 | ns |
| Pex16 KO | AMP | 0.001 | 0.005 | ** |
| Control+LPD | AMP | 0.525 | 0.630 | ns |
| Pex16 KO+LPD | ADP | 0.024 | 0.143 | ns |
| Pex16 KO | ADP | 0.431 | 0.517 | ns |
| Control+LPD | ADP | 0.784 | 0.784 | ns |
| Pex16 KO+LPD | ATP | 0.789 | 0.912 | ns |
| Pex16 KO | ATP | 0.663 | 0.912 | ns |
| Control+LPD | ATP | 0.519 | 0.912 | ns |
| Pex16 KO+LPD | GMP | 0.131 | 0.173 | ns |
| Pex16 KO | GMP | 0.001 | 0.006 | ** |
| Control+LPD | GMP | 0.134 | 0.173 | ns |
| Pex16 KO+LPD | GDP | 0.010 | 0.057 | ns |
| Pex16 KO | GDP | 0.693 | 0.693 | ns |
| Control+LPD | GDP | 0.444 | 0.533 | ns |
| Pex16 KO+LPD | GTP | 0.822 | 0.962 | ns |
| Pex16 KO | GTP | 0.337 | 0.962 | ns |
| Control+LPD | GTP | 0.633 | 0.962 | ns |
| Pex16 KO+LPD | UMP | 0.174 | 0.659 | ns |
| Pex16 KO | UMP | 0.933 | 0.999 | ns |
| Control+LPD | UMP | 0.487 | 0.730 | ns |
| Pex16 KO+LPD | UDP | 0.112 | 0.362 | ns |
| Pex16 KO | UDP | 0.121 | 0.362 | ns |
| Control+LPD | UDP | 0.731 | 0.731 | ns |
| Pex16 KO+LPD | UTP | 0.426 | 0.638 | ns |
| Pex16 KO | UTP | 0.719 | 0.719 | ns |
| Control+LPD | UTP | 0.633 | 0.719 | ns |
| Pex16 KO+LPD | cyclic.ADP.ribose | 0.002 | 0.012 | * |
| Pex16 KO | cyclic.ADP.ribose | 0.285 | 0.285 | ns |
| Control+LPD | cyclic.ADP.ribose | 0.026 | 0.052 | ns |
| Pex16 KO+LPD | cyclic.AMP | 0.008 | 0.025 | * |
| Pex16 KO | cyclic.AMP | 0.063 | 0.063 | ns |
| Control+LPD | cyclic.AMP | 0.018 | 0.027 | * |
| Pex16 KO+LPD | X6.P.Gluconate | 0.004 | 0.012 | * |
| Pex16 KO | X6.P.Gluconate | 0.118 | 0.178 | ns |
| Control+LPD | X6.P.Gluconate | 0.004 | 0.012 | * |
| Pex16 KO+LPD | Acetylglucosamine.1P | 0.974 | 0.995 | ns |
| Pex16 KO | Acetylglucosamine.1P | 0.137 | 0.412 | ns |
| Control+LPD | Acetylglucosamine.1P | 0.897 | 0.995 | ns |
| Pex16 KO+LPD | Acetyl.Phosphate | 0.615 | 0.868 | ns |
| Pex16 KO | Acetyl.Phosphate | 0.036 | 0.217 | ns |
| Control+LPD | Acetyl.Phosphate | 0.944 | 0.944 | ns |
| Pex16 KO+LPD | ADP.Glucose | 0.006 | 0.035 | * |
| Pex16 KO | ADP.Glucose | 0.320 | 0.320 | ns |
| Control+LPD | ADP.Glucose | 0.174 | 0.209 | ns |
| Pex16 KO+LPD | X2.3.bisP.Glycerate | 0.222 | 0.267 | ns |
| Pex16 KO | X2.3.bisP.Glycerate | 0.021 | 0.064 | ns |
| Control+LPD | X2.3.bisP.Glycerate | 0.016 | 0.064 | ns |
| Pex16 KO+LPD | DHAP | 0.348 | 0.695 | ns |
| Pex16 KO | DHAP | 0.047 | 0.280 | ns |
| Control+LPD | DHAP | 0.951 | 0.951 | ns |
| Pex16 KO+LPD | Total.Frucose.bisP.Glucose.1.6.bisP | 0.189 | 0.491 | ns |
| Pex16 KO | Total.Frucose.bisP.Glucose.1.6.bisP | 0.752 | 0.752 | ns |
| Control+LPD | Total.Frucose.bisP.Glucose.1.6.bisP | 0.560 | 0.672 | ns |
| Pex16 KO+LPD | Fructose.6P | 0.514 | 0.772 | ns |
| Pex16 KO | Fructose.6P | 0.121 | 0.728 | ns |
| Control+LPD | Fructose.6P | 0.719 | 0.863 | ns |
| Pex16 KO+LPD | Glucosamine.6P | 0.118 | 0.550 | ns |
| Pex16 KO | Glucosamine.6P | 0.821 | 0.821 | ns |
| Control+LPD | Glucosamine.6P | 0.353 | 0.550 | ns |
| Pex16 KO+LPD | Glycerate.2..or.3.P | 0.401 | 0.761 | ns |
| Pex16 KO | Glycerate.2..or.3.P | 0.843 | 0.843 | ns |
| Control+LPD | Glycerate.2..or.3.P | 0.445 | 0.761 | ns |
| Pex16 KO+LPD | Glycerol.3.P | 0.304 | 0.656 | ns |
| Pex16 KO | Glycerol.3.P | 0.437 | 0.656 | ns |
| Control+LPD | Glycerol.3.P | 0.353 | 0.656 | ns |
| Pex16 KO+LPD | PEP | 0.845 | 0.845 | ns |
| Pex16 KO | PEP | 0.723 | 0.845 | ns |
| Control+LPD | PEP | 0.714 | 0.845 | ns |
| Pex16 KO+LPD | Phosphocreatine | 0.671 | 0.758 | ns |
| Pex16 KO | Phosphocreatine | 0.724 | 0.758 | ns |
| Control+LPD | Phosphocreatine | 0.089 | 0.533 | ns |
| Pex16 KO+LPD | Ribulose.1.5.bisP | 0.054 | 0.082 | ns |
| Pex16 KO | Ribulose.1.5.bisP | 0.846 | 0.846 | ns |
| Control+LPD | Ribulose.1.5.bisP | 0.199 | 0.238 | ns |
| Pex16 KO+LPD | Ribulose.5P | 0.195 | 0.418 | ns |
| Pex16 KO | Ribulose.5P | 0.058 | 0.345 | ns |
| Control+LPD | Ribulose.5P | 0.939 | 0.939 | ns |
| Pex16 KO+LPD | Sedoheptulose.7P | 0.026 | 0.158 | ns |
| Pex16 KO | Sedoheptulose.7P | 0.264 | 0.793 | ns |
| Control+LPD | Sedoheptulose.7P | 0.487 | 0.893 | ns |
| Pex16 KO+LPD | UDP.Glucose | 0.049 | 0.295 | ns |
| Pex16 KO | UDP.Glucose | 0.463 | 0.463 | ns |
| Control+LPD | UDP.Glucose | 0.311 | 0.374 | ns |
| Pex16 KO+LPD | Acetyl.CoA | 0.002 | 0.007 | ** |
| Pex16 KO | Acetyl.CoA | 0.902 | 0.902 | ns |
| Control+LPD | Acetyl.CoA | 0.014 | 0.017 | * |
| Pex16 KO+LPD | Hs.CoA | 0.000 | 0.001 | *** |
| Pex16 KO | Hs.CoA | 0.047 | 0.047 | * |
| Control+LPD | Hs.CoA | 0.001 | 0.001 | ** |
| Pex16 KO+LPD | Malonyl.CoA | 0.100 | 0.300 | ns |
| Pex16 KO | Malonyl.CoA | 0.027 | 0.164 | ns |
| Control+LPD | Malonyl.CoA | 0.669 | 0.972 | ns |
| Pex16 KO+LPD | Succinyl.CoA | 0.000 | 0.000 | *** |
| Pex16 KO | Succinyl.CoA | 0.000 | 0.000 | *** |
| Control+LPD | Succinyl.CoA | 0.000 | 0.000 | *** |
| Pex16 KO+LPD | NAD. | 0.001 | 0.006 | ** |
| Pex16 KO | NAD. | 0.045 | 0.053 | ns |
| Control+LPD | NAD. | 0.053 | 0.053 | ns |
| Pex16 KO+LPD | NADH | 0.925 | 0.925 | ns |
| Pex16 KO | NADH | 0.084 | 0.126 | ns |
| Control+LPD | NADH | 0.788 | 0.925 | ns |
| Pex16 KO+LPD | NADP. | 0.483 | 0.580 | ns |
| Pex16 KO | NADP. | 0.237 | 0.361 | ns |
| Control+LPD | NADP. | 0.783 | 0.783 | ns |
| Pex16 KO+LPD | NADPH | 0.000 | 0.000 | *** |
| Pex16 KO | NADPH | 0.431 | 0.431 | ns |
| Control+LPD | NADPH | 0.000 | 0.001 | *** |
| Pex16 KO+LPD | Erythrose.4P | 0.161 | 0.161 | ns |
| Pex16 KO | Erythrose.4P | 0.099 | 0.119 | ns |
| Control+LPD | Erythrose.4P | 0.033 | 0.066 | ns |
| Pex16 KO+LPD | Glucose | 0.000 | 0.000 | *** |
| Pex16 KO | Glucose | 0.019 | 0.019 | * |
| Control+LPD | Glucose | 0.000 | 0.000 | *** |
| Pex16 KO+LPD | Glucose.6P | 0.003 | 0.005 | ** |
| Pex16 KO | Glucose.6P | 0.257 | 0.257 | ns |
| Control+LPD | Glucose.6P | 0.004 | 0.005 | ** |
| Pex16 KO+LPD | Glycerylaldehyde.3P | 0.002 | 0.002 | ** |
| Pex16 KO | Glycerylaldehyde.3P | 0.527 | 0.527 | ns |
| Control+LPD | Glycerylaldehyde.3P | 0.001 | 0.002 | ** |
| Pex16 KO+LPD | Mannose.6P | 0.011 | 0.013 | * |
| Pex16 KO | Mannose.6P | 0.547 | 0.547 | ns |
| Control+LPD | Mannose.6P | 0.007 | 0.010 | * |
| Pex16 KO+LPD | Ribose.5P | 0.000 | 0.001 | ** |
| Pex16 KO | Ribose.5P | 0.009 | 0.009 | ** |
| Control+LPD | Ribose.5P | 0.001 | 0.001 | ** |
| Pex16 KO+LPD | a.Hydroxyglutaric.acid | 0.013 | 0.029 | * |
| Pex16 KO | a.Hydroxyglutaric.acid | 0.086 | 0.103 | ns |
| Control+LPD | a.Hydroxyglutaric.acid | 0.010 | 0.029 | * |
| Pex16 KO+LPD | a.Ketoglutaric.acid | 0.001 | 0.006 | ** |
| Pex16 KO | a.Ketoglutaric.acid | 0.086 | 0.103 | ns |
| Control+LPD | a.Ketoglutaric.acid | 0.002 | 0.007 | ** |
| Pex16 KO+LPD | Citric.acid | 0.390 | 0.818 | ns |
| Pex16 KO | Citric.acid | 0.681 | 0.818 | ns |
| Control+LPD | Citric.acid | 0.610 | 0.818 | ns |
| Pex16 KO+LPD | Fumaric.acid | 0.663 | 0.796 | ns |
| Pex16 KO | Fumaric.acid | 0.973 | 0.973 | ns |
| Control+LPD | Fumaric.acid | 0.261 | 0.796 | ns |
| Pex16 KO+LPD | Glycolic.acid | 0.588 | 0.882 | ns |
| Pex16 KO | Glycolic.acid | 0.908 | 0.915 | ns |
| Control+LPD | Glycolic.acid | 0.915 | 0.915 | ns |
| Pex16 KO+LPD | Isocitric.acid | 0.157 | 0.943 | ns |
| Pex16 KO | Isocitric.acid | 0.950 | 0.950 | ns |
| Control+LPD | Isocitric.acid | 0.806 | 0.950 | ns |
| Pex16 KO+LPD | Latic.acid | 0.014 | 0.036 | * |
| Pex16 KO | Latic.acid | 0.608 | 0.729 | ns |
| Control+LPD | Latic.acid | 0.018 | 0.036 | * |
| Pex16 KO+LPD | Malic.acid | 0.525 | 0.738 | ns |
| Pex16 KO | Malic.acid | 0.951 | 0.951 | ns |
| Control+LPD | Malic.acid | 0.233 | 0.738 | ns |
| Pex16 KO+LPD | Pyruvic.acid | 0.884 | 0.922 | ns |
| Pex16 KO | Pyruvic.acid | 0.922 | 0.922 | ns |
| Control+LPD | Pyruvic.acid | 0.636 | 0.922 | ns |
| Pex16 KO+LPD | Succinic.acid | 0.856 | 0.872 | ns |
| Pex16 KO | Succinic.acid | 0.054 | 0.323 | ns |
| Control+LPD | Succinic.acid | 0.858 | 0.872 | ns |

*FDR corrected p-values, *p<0.05, **p<0.01, ***p<0.001, versus wild-type mice fed a 18% protein diet.
